# Supplementary material for: Recurrent evolution of ligand-binding domain multiplicity fine-tunes TGFβ signaling in vertebrates
Source: Nat Commun. 2026 May 19;17:4458. doi: 10.1038/s41467-026-73340-5 (PMC13187141; doi:10.1038/s41467-026-73340-5)
Supplement: Supplementary file 1 — Supplementary Information [file 41467_2026_73340_MOESM1_ESM.pdf]

Supplementary Information

Recurrent evolution of ligand-binding domain multiplicity fine-tunes TGFβ signaling in vertebrates

Jerome Jatzlau[1, *, ✉], Michael Trumpp[1,2, *], Julia Kühlwein[1], Leon Obendorf[1,2], Yao Le[3], Heiner Kuhl[4,5], Marco Preussner[1], Paul Mendez[1], Hendrik Burkert[1], Wiktor Burdzinski[1,6], Stefan Mundlos[7], Christoph Winkler[3], Matthias Stöck[4], Petra Knaus[1,6, ✉]

[1] Freie Universität Berlin, Institute of Chemistry and Biochemistry - Biochemistry, Berlin, Germany.

[2] International Max Planck Research School for Biology and Computation, Berlin, Germany

[3] Department of Biological Sciences and Centre for Bioimaging Sciences, National University of Singapore, Singapore, Singapore.

[4] Department of Fish Biology, Fisheries and Aquaculture, Leibniz Institute of Freshwater Ecology and Inland Fisheries, Berlin, Germany.

[5] Ecotoxicological Laboratory, German Environment Agency (UBA), Berlin, Germany.

[6] Berlin-Brandenburg School for Regenerative Therapies (BSRT), Berlin, Germany.

[7] Max Planck Institute for Molecular Genetics, Berlin, Germany.

✉ Corresponding author

* Equal contributing authors

These authors contributed equally*: Jerome Jatzlau, Michael Trumpp. Correspondence and requests for materials should be addressed to J.J. (email: jerome.jatzlau@fu-berlin.de) or to P.K. (email: petra.knaus@fu-berlin.de).

Inventory:

Supplementary Figure 1: TGFBR2 LBD duplication independent of allotetraploid chromosomal evolution in *Cyprinus carpio* and *Xenopus laevis*.
Supplementary Figure 2: Genetic architecture of duplicated LBDs of TGFβ receptor family members.
Supplementary Figure 3: LBD sequence identity comparison of ACVR1 and BMPR2 orthologs.
Supplementary Figure 4: Inner LBD conveys Activin A binding competence of LBD multimerized BMPR2 orthologs.
Supplementary Figure 5: Representative LSBA images of Activin A bound to gnBmpr2a variants.
Supplementary Figure 6: LBD sequence identity comparison of TGFBR2 orthologs.
Supplementary Figure 7: Sequence alignment of multimerized TGFBR2 LBDs and TGFβ1 orthologs.
Supplementary Figure 8: Evolutionary divergence of duplicated LBD domains relative to species divergence times.
Supplementary Figure 9: Functional implications of LBD multiplication in TGFBR2 orthologs of *Cyprinus carpio* and *Xenopus laevis*.
Supplementary Figure 10: *In silico* binding analysis of TGFBR2 orthologs with respective animal TGFβ1.
Supplementary Figure 11: *In silico* binding analysis of ray-finned fish TGFBR2 orthologs with human and respective animal TGFβ1.
Supplementary Figure 12: Representative LSBA images of TGFβ1 bound to dTgfbr2a/b variants.
Supplementary Figure 13: Representative LSBA images of TGFβ1 bound to cTgfbr2a variants.
Supplementary Figure 14: Representative LSBA images of TGFβ1 bound to xlTgfbr2.L variants.
Supplementary Figure 15: Representative LSBA images of TGFβ1 bound to eTGFBR2 variants.
Supplementary Figure 16: Representative LSBA images of TGFβ1 bound to gTGFBR2 variants.
Supplementary Figure 17: Linker architecture in TGFBR2 and BMPR2 variants.
Supplementary Figure 18: Representative LSBA images of TGFβ1 bound to hTGFBR2 variants.
Supplementary Figure 19: Alternative splice variants of TGFBR2 in horse and chicken.
Supplementary Figure 20: Coinjected membrane bound PMT-mEGFP expression control in zebrafish embryos.
Supplementary Figure 21: Western blot expression controls of Halo-tagged receptor constructs.
Supplementary Table 1: N-terminally tagged HaloTag expression constructs

Supplementary Table 2: Oligonucleotides used for molecular cloning of LBD-Halo constructs

Supplementary Figure 22: Uncropped Western blots

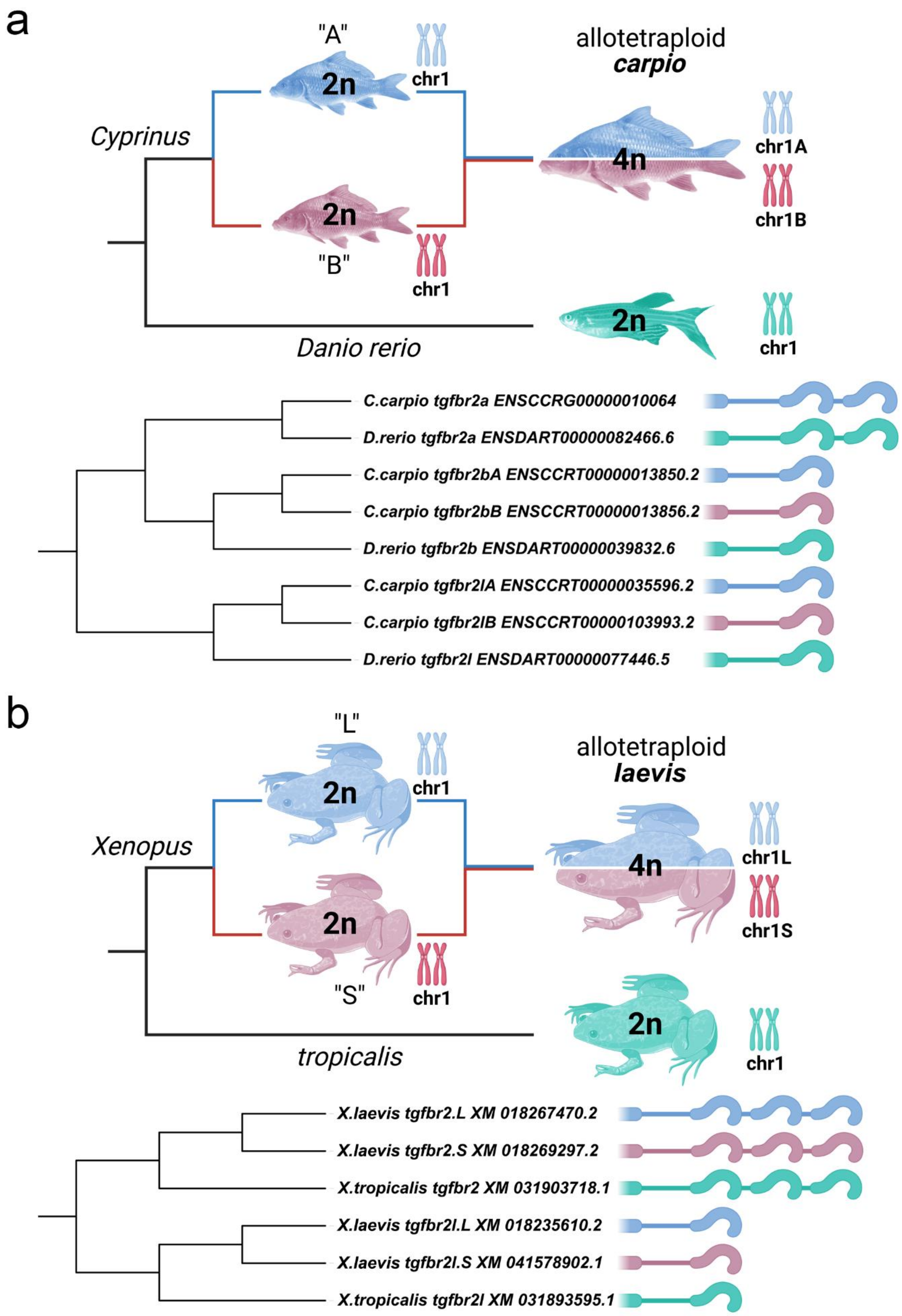

Supplementary Figure 1: TGFBR2 LBD duplication independent of allotetraploid chromosomal evolution in *Cyprinus carpio* and *Xenopus laevis*. (**a, top**) Phylogenetic depiction of chromosomal evolution in the Cyprinus lineage, illustrating the allopolyploid origin of *Cyprinus carpio* (4n) from two diploid ancestors ("A" and "B"). Each ancestor contributed a

set of chromosomes (chr1A in blue, chr1B in red) leading to a tetraploid *C. carpio* with both ancestral chromosome sets. *Danio rerio* is depicted with its diploid structure (2n), maintaining a single chromosome set (chr1). (**Bottom)** Phylogenetic tree showing the relationship of tgfbr2a gene variants between *C. carpio* and *D. rerio*. Structural variations in ligand-binding domains are illustrated beside each gene. (**b, top**) Phylogenetic depiction of chromosomal evolution in the Xenopus lineage, illustrating the allopolyploid origin of *Xenopus laevis* (4n) from two diploid ancestors (“L" and “S"). Each ancestor contributed a set of chromosomes (chr1L in blue, chr1S in red) leading to a tetraploid *X. laevis* with both ancestral chromosome sets. *Xenopus tropicalis* is depicted with its diploid structure (2n), maintaining a single chromosome set (chr1). (**Bottom)** Phylogenetic tree showing the relationship of tgfbr2 gene variants between X*. laevis and X. tropicalis.* Structural variations in ligand-binding domains are illustrated beside each gene. (**a, b**) Schematics created in BioRender. Trumpp, M. (2026), https://BioRender.com/8p64dhh.

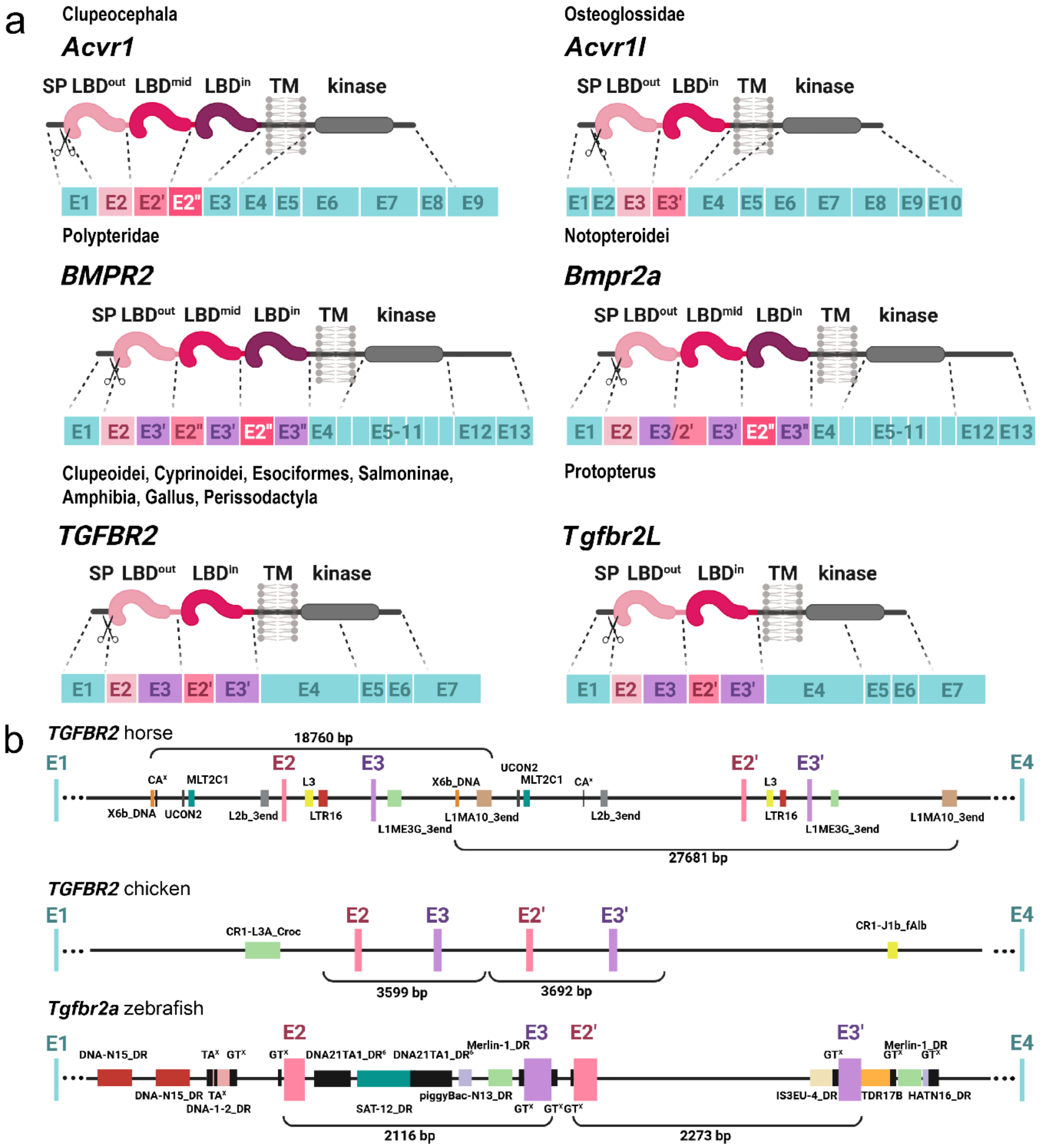

Supplementary Figure 2: Genetic architecture of duplicated LBDs of TGFβ receptor family members. (**a**) Schematic representation of receptor gene exon composition of Acvr1, Acvr1L, BMPR2, Bmpr2a, TGFBR2 and TGFBR2L. (**b**) Genomic organization of TGFBR2 intronic sites flanking exons encoding in horse, chicken, and Tgfbr2a in zebrafish, including annotations size and common repetitive elements. (**a, b**) Schematics created in BioRender. Trumpp, M. (2026), https://BioRender.com/8p64dhh.

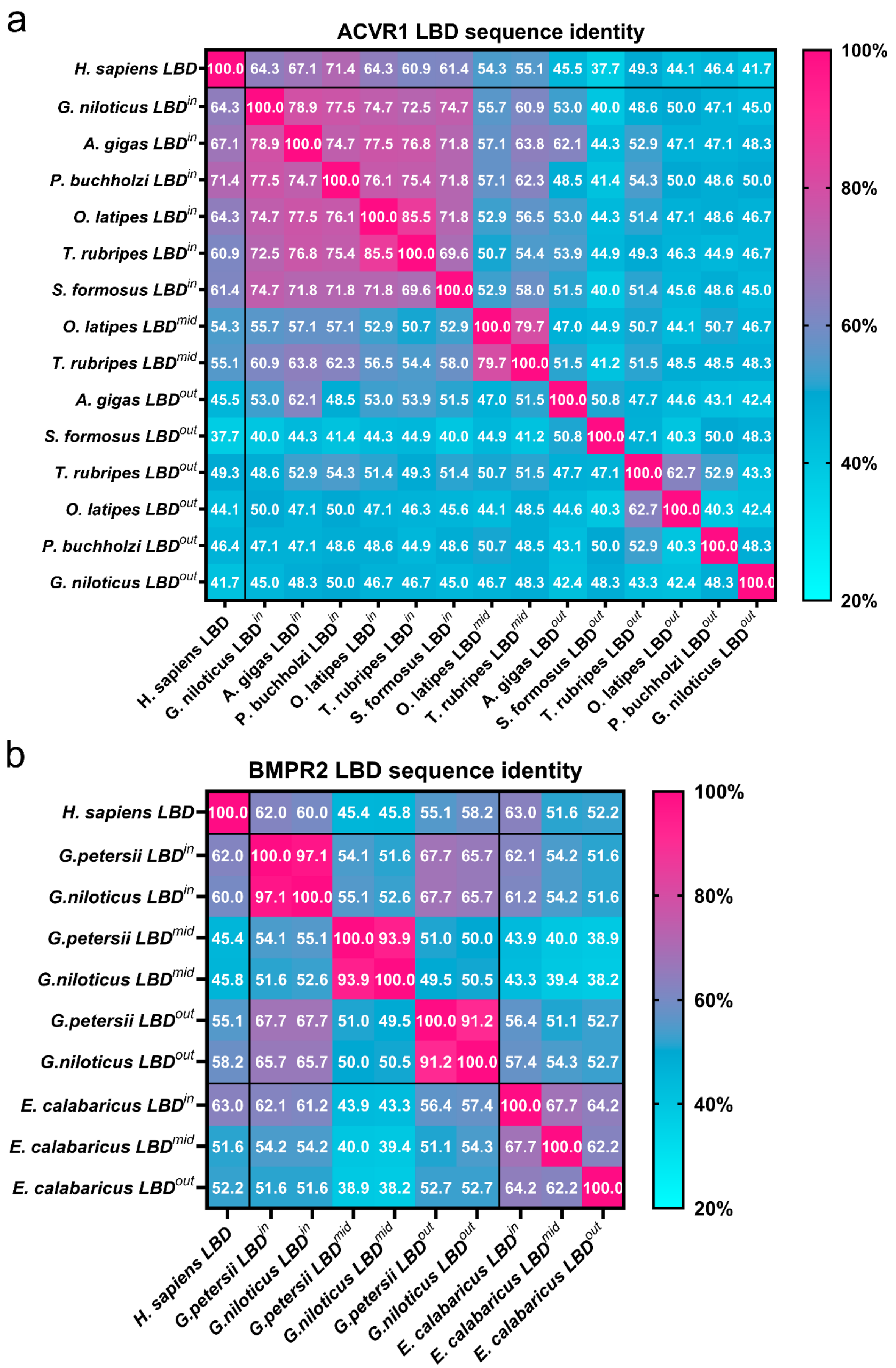
a
ACVR1 LBD sequence identity
H. sapiens LBD 100.0 64.3 67.1 71.4 64.3 60.9 61.4 54.3 55.1 45.5 37.7 49.3 44.1 46.4 41.7
G. niloticus LBD^in 64.3 100.0 78.9 77.5 74.7 72.5 74.7 55.7 60.9 53.0 40.0 48.6 50.0 47.1 45.0
A. gigas LBD^in 67.1 78.9 100.0 74.7 77.5 76.8 71.8 57.1 63.8 62.1 44.3 52.9 47.1 47.1 48.3
P. buchholzi LBD^in 71.4 77.5 74.7 100.0 76.1 75.4 71.8 57.1 62.3 48.5 41.4 54.3 50.0 48.6 50.0
O. latipes LBD^in 64.3 74.7 77.5 76.1 100.0 85.5 71.8 52.9 56.5 53.0 44.3 51.4 47.1 48.6 46.7
T. rubripes LBD^in 60.9 72.5 76.8 75.4 85.5 100.0 69.6 50.7 54.4 53.9 44.9 49.3 46.3 44.9 46.7
S. formosus LBD^in 61.4 74.7 71.8 71.8 71.8 69.6 100.0 52.9 58.0 51.5 40.0 51.4 45.6 48.6 45.0
O. latipes LBD^mid 54.3 55.7 57.1 57.1 52.9 50.7 52.9 100.0 79.7 47.0 44.9 50.7 44.1 50.7 46.7
T. rubripes LBD^mid 55.1 60.9 63.8 62.3 56.5 54.4 58.0 79.7 100.0 51.5 41.2 51.5 48.5 48.5 48.3
A. gigas LBD^out 45.5 53.0 62.1 48.5 53.0 53.9 51.5 47.0 51.5 100.0 50.8 47.7 44.6 43.1 42.4
S. formosus LBD^out 37.7 40.0 44.3 41.4 44.3 44.9 40.0 44.9 41.2 50.8 100.0 47.1 40.3 50.0 48.3
T. rubripes LBD^out 49.3 48.6 52.9 54.3 51.4 49.3 51.4 50.7 51.5 47.7 47.1 100.0 62.7 52.9 43.3
O. latipes LBD^out 44.1 50.0 47.1 50.0 47.1 46.3 45.6 44.1 48.5 44.6 40.3 62.7 100.0 40.3 42.4
P. buchholzi LBD^out 46.4 47.1 47.1 48.6 48.6 44.9 48.6 50.7 48.5 43.1 50.0 52.9 40.3 100.0 48.3
G. niloticus LBD^out 41.7 45.0 48.3 50.0 46.7 46.7 45.0 46.7 48.3 42.4 48.3 43.3 42.4 48.3 100.0
100%
80%
60%
40%
20%
b
BMPR2 LBD sequence identity
H. sapiens LBD 100.0 62.0 60.0 45.4 45.8 55.1 58.2 63.0 51.6 52.2
G.petersii LBD^in 62.0 100.0 97.1 54.1 51.6 67.7 65.7 62.1 54.2 51.6
G.niloticus LBD^in 60.0 97.1 100.0 55.1 52.6 67.7 65.7 61.2 54.2 51.6
G.petersii LBD^mid 45.4 54.1 55.1 100.0 93.9 51.0 50.0 43.9 40.0 38.9
G.niloticus LBD^mid 45.8 51.6 52.6 93.9 100.0 49.5 50.5 43.3 39.4 38.2
G.petersii LBD^out 55.1 67.7 67.7 51.0 49.5 100.0 91.2 56.4 51.1 52.7
G.niloticus LBD^out 58.2 65.7 65.7 50.0 50.5 91.2 100.0 57.4 54.3 52.7
E. calabaricus LBD^in 63.0 62.1 61.2 43.9 43.3 56.4 57.4 100.0 67.7 64.2
E. calabaricus LBD^mid 51.6 54.2 54.2 40.0 39.4 51.1 54.3 67.7 100.0 62.2
E. calabaricus LBD^out 52.2 51.6 51.6 38.9 38.2 52.7 52.7 64.2 62.2 100.0
100%
80%
60%
40%
20%

Supplementary Figure 3: LBD sequence identity comparison of ACVR1 and BMPR2 orthologs. (**a**) ACVR1 LBD sequence identity matrix comparing all LBD ACVR1 variants of indicated species. (**b**) BMPR2 LBD sequence identity matrix comparing all LBD BMPR2 variants of indicated species with each other.

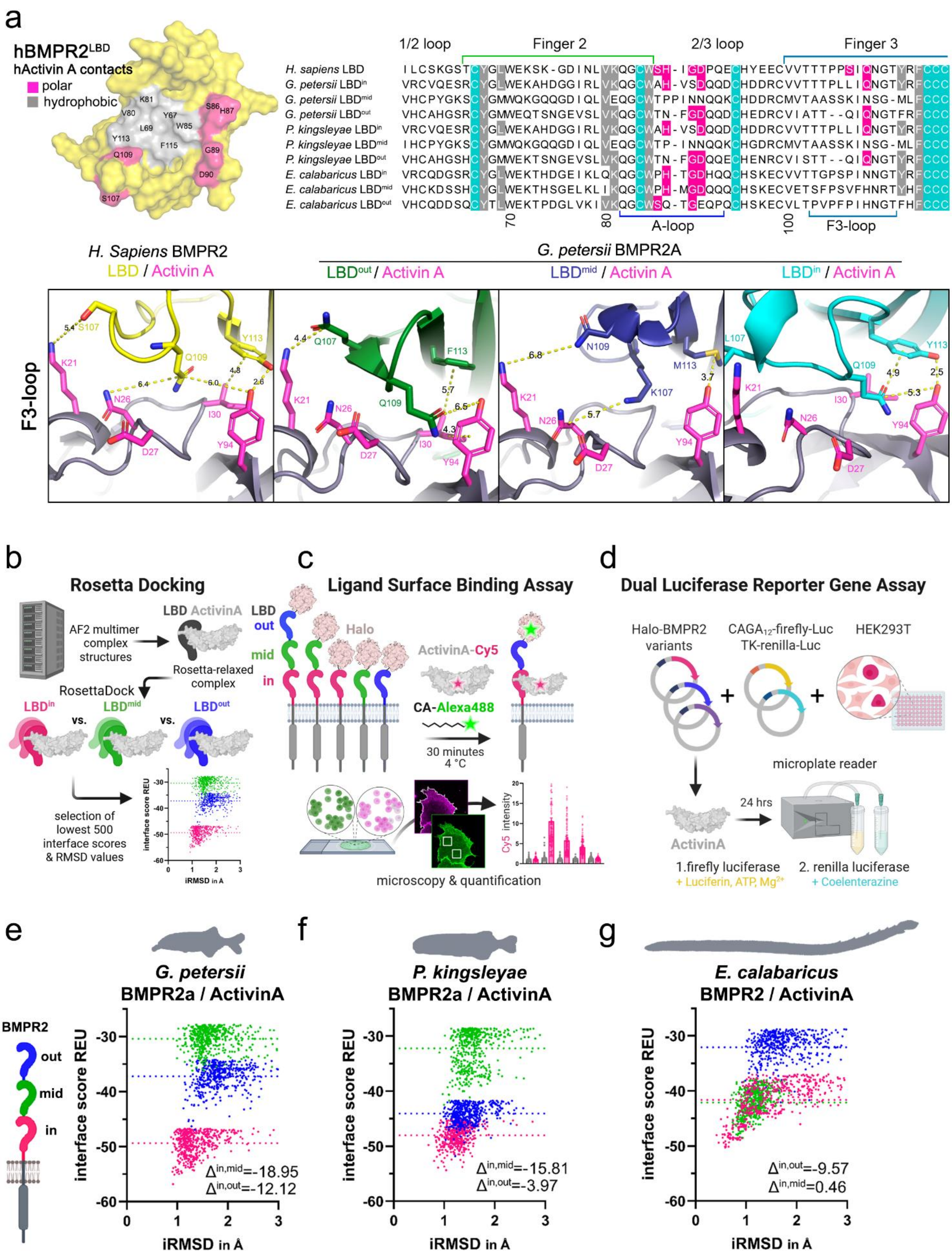

Supplementary Figure 4: Inner LBD conveys Activin A binding competence of LBD multimerized BMPR2 orthologs. (**a**) Molecular surface of hBMPR2 LBD showing the hActivin A interface of a rosetta-docked AlphaFold2-multimer structure prediction. Residues forming

polar or hydrophobic contacts are colored in pink and gray, respectively (**left**). Sequence alignment of BMPR2 LBDs of *Homo Sapiens*, *G. petersii*, *G.niloticus* and *E. calabaricus* LBDs with hActivin A contact residues and critical backbone cysteins (teal) highlighted; interaction sites F3-loop and A-loop are indicated with colored lines (**right**). (**a, lower**) Cartoon and stick representation of respective animal receptor LBD interaction with hActivin A (A-loop) based on AlphaFold2-multimer models docked to hActivin A using Rosetta docking protocols. PyMOL was used for image representation. (**b**) Schematic representation of *in silico* binding analysis workflow using Rosetta docking. Alphafold (AF2 multimer) LBD-Activin A complex structures are generated, followed by initial coordinate constrained relaxation to obtain optimized H-bonds. Docking of LBDs is then performed through RosettaScripts (n=2500). Interface score (REU) versus iRMSD plots identify the top 500 models with the lowest scores, indicating different ligand binding capabilites. (**c**) Illustration of LSBA for visualization and quantification of fluorescent hActivin A binding on COS-7 cells expressing respective Halo-tagged BMPR2 receptor constructs. (**d**) Schematic representation of dual luciferase reporter gene assay used to assess Activin A signaling activity through Halo-tagged BMRP2 receptor orthologs. HEK293T cells are co-transfected with respective receptor variant, $CAGA_{12}$-firefly-Luc, and TK-renilla-Luc plasmids. Following Activin A treatment for 24 hours, Firefly luciferase activity (indicative of Activin A pathway activation) and Renilla luciferase activity (normalization control) are measured. *In silico* binding analysis of (**e**) *G. petersii* (**f**) *P. kingsleyae* (**g**) *E. calabaricus* Activin A interactions with its respective single Bmpr2a domains obtained via Rosetta Docking, depicted as interface score (REU) in relation to interface root mean square deviation (iRMSD) in angstroms. (**right**) Illustration of respective animal BMPR2a 3 LBD receptor domain structure as reference, highlighting the inner (pink), middle (green) and outer (blue) ligand binding domain. REU differences of out and mid LBD mean are calculated against inner LBD mean value. Source data are provided as a Source Data file. (**b–d, e**) Schematics created in BioRender. Trumpp, M. (2026), https://BioRender.com/8p64dhh.

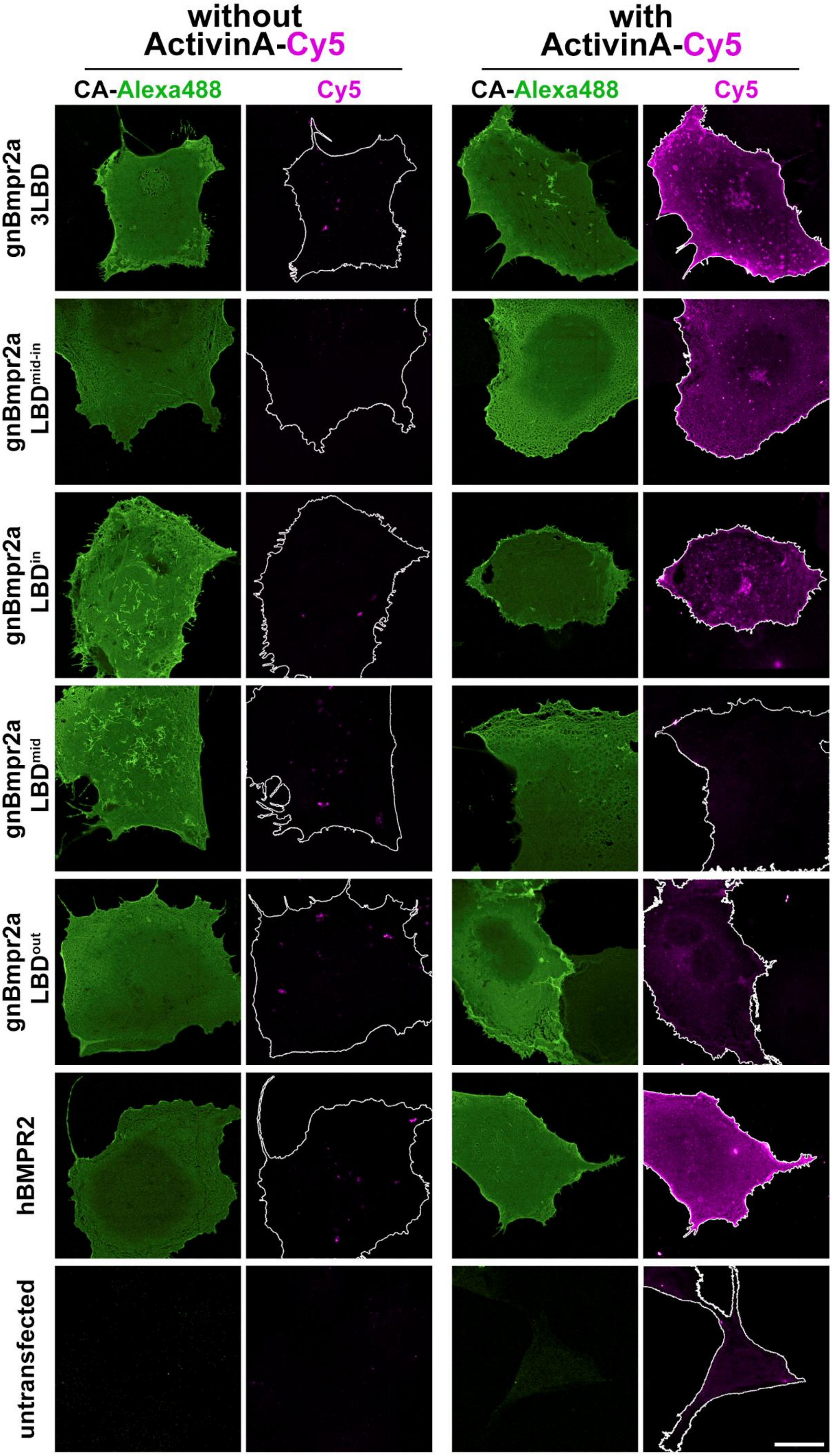

without ActivinA-Cy5
with ActivinA-Cy5
CA-Alexa488
Cy5
CA-Alexa488
Cy5
gnBmpr2a 3LBD
gnBmpr2a LBD^mid-in
gnBmpr2a LBD^in
gnBmpr2a LBD^mid
gnBmpr2a LBD^out
hBMPR2
untransfected

Supplementary Figure 5: Representative LSBA images of Activin A bound to gnBmpr2a variants. Representative confocal microscopy images of LSBA for unstimulated and hActivinA-Cy5 stimulated COS-7 cells expressing *Gnathonemus petersii* receptor variants (gnBmpr2a (3LBD), gnBmpr2a-LBD^{midin}, gnBmpr2a-LBD^{in}, gnBmpr2a-LBD^{mid}, gnBmpr2a-LBD^{out}) or hBMPR2 or untransfected control. Scale bar ≙ 20 µm.

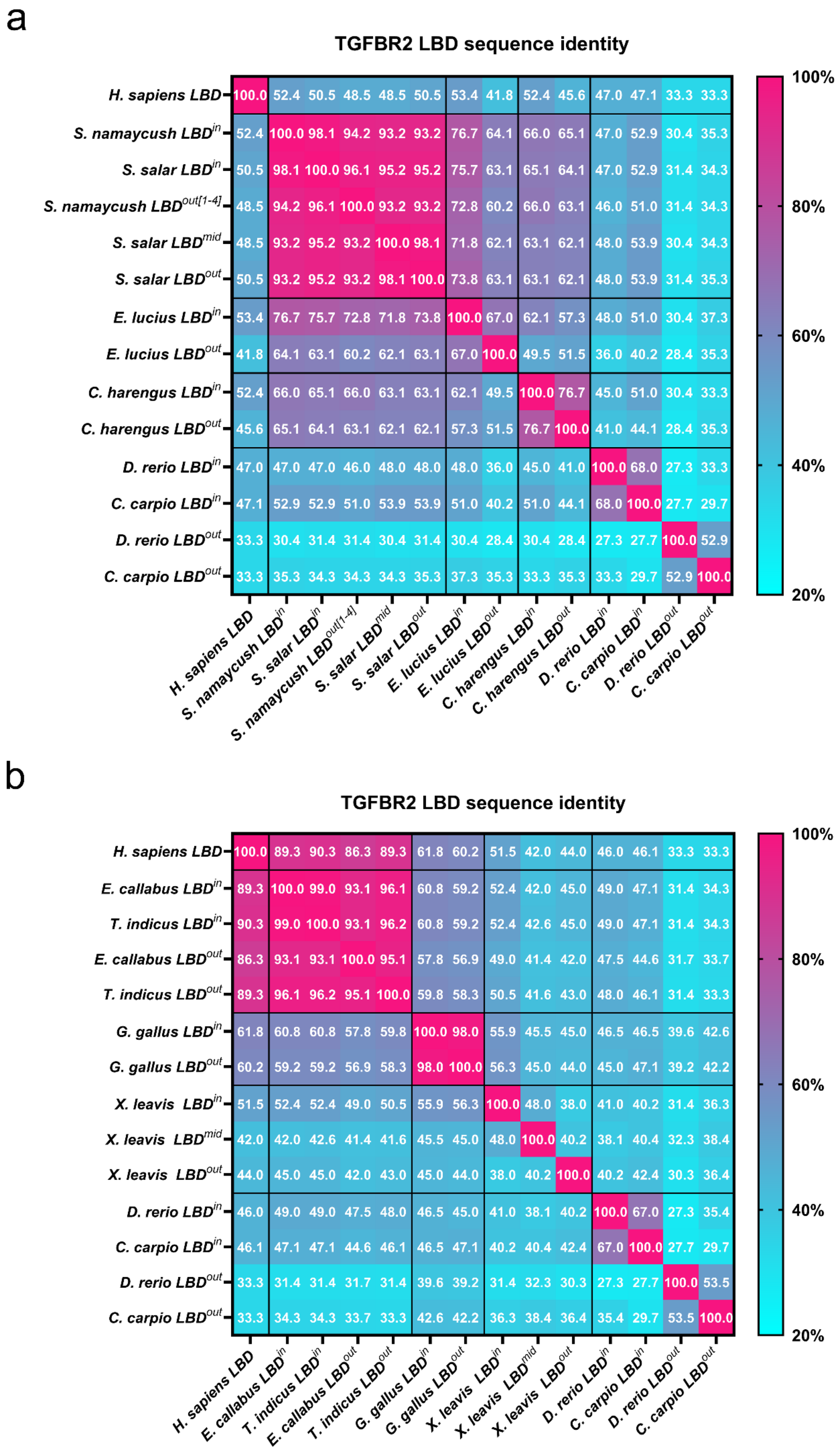
a
TGFBR2 LBD sequence identity
H. sapiens LBD 100.0 52.4 50.5 48.5 48.5 50.5 53.4 41.8 52.4 45.6 47.0 47.1 33.3 33.3
S. namaycush LBDin 52.4 100.0 98.1 94.2 93.2 93.2 76.7 64.1 66.0 65.1 47.0 52.9 30.4 35.3
S. salar LBDin 50.5 98.1 100.0 96.1 95.2 95.2 75.7 63.1 65.1 64.1 47.0 52.9 31.4 34.3
S. namaycush LBDout[1-4] 48.5 94.2 96.1 100.0 93.2 93.2 72.8 60.2 66.0 63.1 46.0 51.0 31.4 34.3
S. salar LBDmid 48.5 93.2 95.2 93.2 100.0 98.1 71.8 62.1 63.1 62.1 48.0 53.9 30.4 34.3
S. salar LBDout 50.5 93.2 95.2 93.2 98.1 100.0 73.8 63.1 63.1 62.1 48.0 53.9 31.4 35.3
E. lucius LBDin 53.4 76.7 75.7 72.8 71.8 73.8 100.0 67.0 62.1 57.3 48.0 51.0 30.4 37.3
E. lucius LBDout 41.8 64.1 63.1 60.2 62.1 63.1 67.0 100.0 49.5 51.5 36.0 40.2 28.4 35.3
C. harengus LBDin 52.4 66.0 65.1 66.0 63.1 63.1 62.1 49.5 100.0 76.7 45.0 51.0 30.4 33.3
C. harengus LBDout 45.6 65.1 64.1 63.1 62.1 62.1 57.3 51.5 76.7 100.0 41.0 44.1 28.4 35.3
D. rerio LBDin 47.0 47.0 47.0 46.0 48.0 48.0 48.0 36.0 45.0 41.0 100.0 68.0 27.3 33.3
C. carpio LBDin 47.1 52.9 52.9 51.0 53.9 53.9 51.0 40.2 51.0 44.1 68.0 100.0 27.7 29.7
D. rerio LBDout 33.3 30.4 31.4 31.4 30.4 31.4 30.4 28.4 30.4 28.4 27.3 27.7 100.0 52.9
C. carpio LBDout 33.3 35.3 34.3 34.3 34.3 35.3 37.3 35.3 33.3 35.3 33.3 29.7 52.9 100.0
100%
80%
60%
40%
20%
H. sapiens LBD
S. namaycush LBDin
S. salar LBDin
S. namaycush LBDout[1-4]
S. salar LBDmid
S. salar LBDout
E. lucius LBDin
E. lucius LBDout
C. harengus LBDin
C. harengus LBDout
D. rerio LBDin
C. carpio LBDin
D. rerio LBDout
C. carpio LBDout
b
TGFBR2 LBD sequence identity
H. sapiens LBD 100.0 89.3 90.3 86.3 89.3 61.8 60.2 51.5 42.0 44.0 46.0 46.1 33.3 33.3
E. callabus LBDin 89.3 100.0 99.0 93.1 96.1 60.8 59.2 52.4 42.0 45.0 49.0 47.1 31.4 34.3
T. indicus LBDin 90.3 99.0 100.0 93.1 96.2 60.8 59.2 52.4 42.6 45.0 49.0 47.1 31.4 34.3
E. callabus LBDout 86.3 93.1 93.1 100.0 95.1 57.8 56.9 49.0 41.4 42.0 47.5 44.6 31.7 33.7
T. indicus LBDout 89.3 96.1 96.2 95.1 100.0 59.8 58.3 50.5 41.6 43.0 48.0 46.1 31.4 33.3
G. gallus LBDin 61.8 60.8 60.8 57.8 59.8 100.0 98.0 55.9 45.5 45.0 46.5 46.5 39.6 42.6
G. gallus LBDout 60.2 59.2 59.2 56.9 58.3 98.0 100.0 56.3 45.0 44.0 45.0 47.1 39.2 42.2
X. leavis LBDin 51.5 52.4 52.4 49.0 50.5 55.9 56.3 100.0 48.0 38.0 41.0 40.2 31.4 36.3
X. leavis LBDmid 42.0 42.0 42.6 41.4 41.6 45.5 45.0 48.0 100.0 40.2 38.1 40.4 32.3 38.4
X. leavis LBDout 44.0 45.0 45.0 42.0 43.0 45.0 44.0 38.0 40.2 100.0 40.2 42.4 30.3 36.4
D. rerio LBDin 46.0 49.0 49.0 47.5 48.0 46.5 45.0 41.0 38.1 40.2 100.0 67.0 27.3 35.4
C. carpio LBDin 46.1 47.1 47.1 44.6 46.1 46.5 47.1 40.2 40.4 42.4 67.0 100.0 27.7 29.7
D. rerio LBDout 33.3 31.4 31.4 31.7 31.4 39.6 39.2 31.4 32.3 30.3 27.3 27.7 100.0 53.5
C. carpio LBDout 33.3 34.3 34.3 33.7 33.3 42.6 42.2 36.3 38.4 36.4 35.4 29.7 53.5 100.0
100%
80%
60%
40%
20%
H. sapiens LBD
E. callabus LBDin
T. indicus LBDin
E. callabus LBDout
T. indicus LBDout
G. gallus LBDin
G. gallus LBDout
X. leavis LBDin
X. leavis LBDmid
X. leavis LBDout
D. rerio LBDin
C. carpio LBDin
D. rerio LBDout
C. carpio LBDout

Supplementary Figure 6: LBD sequence identity comparison of TGFBR2 orthologs. (**a**) TGFBR2 LBD sequence identity matrix comparing all LBD TGFBR2 variants of human and indicated ray finned fish species. (**b**) TGFBR2 LBD sequence identity matrix comparing all LBD TGFBR2 variants of indicated jaw vertebrate as well as Cyprinoidei species.

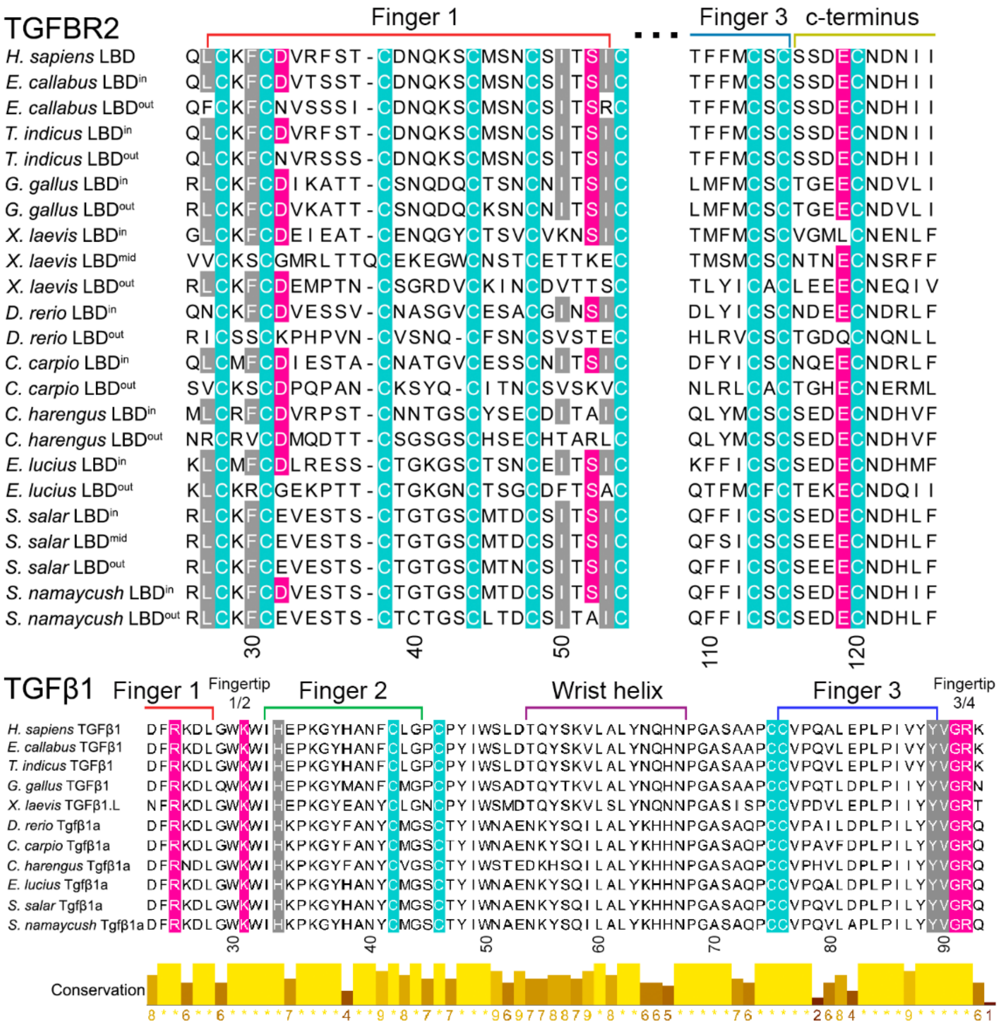

Supplementary Figure 7: Sequence alignment of multimerized TGFBR2 LBDs and TGFβ1 orthologs. TGFBR2 LBD and TGFβ1 ligand sequence alignment of *H. sapiens*, *E. callabus*, *T. indicus*, *G. Gallus*, *X. laevis*, *D. rerio*, *C. carpio*, *C. harengus*, *E. lucius*, *S. salar*, *S. namaycush*; polar receptor:ligand interaction sites are shown in light magenta and hydrophobic interaction sites in grey and highly conserved backbone cysteins in teal. Interaction domains are indicated above.

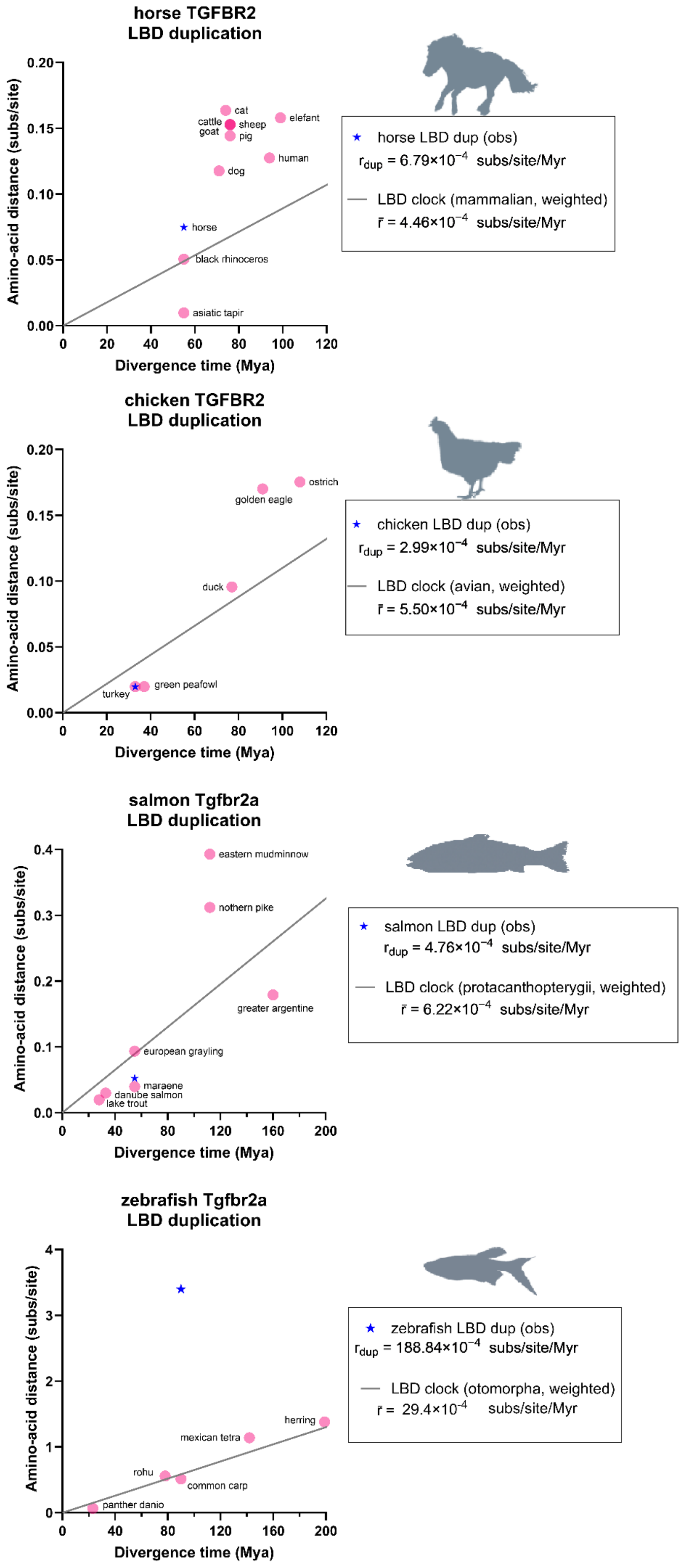
horse TGFBR2
LBD duplication
Amino-acid distance (subs/site)
Divergence time (Mya)
0.00
0.05
0.10
0.15
0.20
0
20
40
60
80
100
120
cat
cattle
goat
sheep
pig
elefant
human
dog
horse
black rhinoceros
asiatic tapir
horse LBD dup (obs)
r_dup = 6.79×10^-4 subs/site/Myr
LBD clock (mammalian, weighted)
r̄ = 4.46×10^-4 subs/site/Myr
chicken TGFBR2
LBD duplication
ostrich
golden eagle
duck
turkey
green peafowl
chicken LBD dup (obs)
r_dup = 2.99×10^-4 subs/site/Myr
LBD clock (avian, weighted)
r̄ = 5.50×10^-4 subs/site/Myr
salmon Tgfbr2a
LBD duplication
0.0
0.1
0.2
0.3
0.4
160
200
eastern mudminnow
nothern pike
greater argentine
european grayling
maraene
danube salmon
lake trout
salmon LBD dup (obs)
r_dup = 4.76×10^-4 subs/site/Myr
LBD clock (protacanthopterygii, weighted)
r̄ = 6.22×10^-4 subs/site/Myr
zebrafish Tgfbr2a
LBD duplication
1
2
3
4
herring
mexican tetra
rohu
common carp
panther danio
zebrafish LBD dup (obs)
r_dup = 188.84×10^-4 subs/site/Myr
LBD clock (otomorpha, weighted)
r̄ = 29.4×10^-4 subs/site/Myr

Supplementary Figure 8: Evolutionary divergence of duplicated LBD domains relative to species divergence times. Each panel shows the relationship between amino-acid distance and species divergence time for duplicated LBDs of TGFBR2/Tgfbr2a in four focal species: horse, chicken, salmon, and zebrafish. Pink circles represent pairwise comparisons between the inner LBD of the focal species and the orthologous inner LBD of other species in the same clade. The gray line depicts the clade-specific molecular clock, obtained by weighted linear regression of amino-acid distance against species divergence time; the resulting substitution rate ($\bar{r}$), expressed as substitutions per site per million years, is shown in each panel. The blue star marks the observed amino-acid divergence between the two duplicated LBD copies within the focal species, and the duplication-specific evolutionary rate ($r_{(}dup_{)}$) is reported in each panel. Source data are provided as a Source Data file.

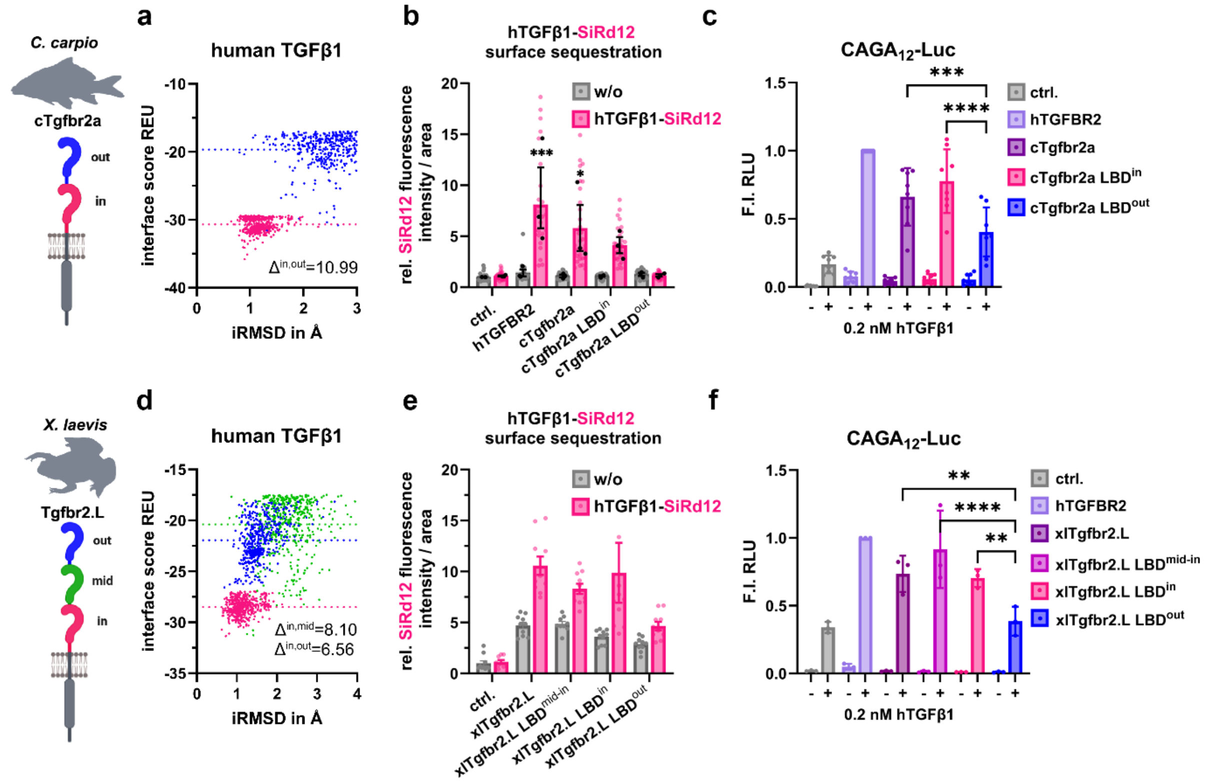

Supplementary Figure 9: Functional implications of LBD multiplication in TGFBR2 orthologs of *Cyprinus carpio* and *Xenopus laevis*. (**a, d)** *in silico* binding analysis via Rosetta docking of hTGFβ1 to inner (pink) and outer LBD (blue) of *C. carpio*, *X. laevis* depicted as interface score (REU) in relation to interface root mean square deviation (iRMSD) in angstroms. REU differences of in and out are calculated by respective mean REU values of each LBD. (**b**) hTGFβ1–SiR-d12 surface binding to C. carpio receptor variants, shown as relative fluorescence intensity per area (± hTGFβ1–SiR-d12). Data are mean ± SEM. n = 3 independent biological replicates (independent experiments; up to 10 cells per experiment averaged). Two-way ANOVA with Dunnett's multiple comparisons test (two-sided; vs ctrl.) was used. Exact P values: hTGFBR2 vs ctrl., p = 0.0009; cTgfbr2a vs ctrl., p = 0.0493; cTgfbr2a LBD^{in} vs ctrl., p = 0.3244. Error bars represent SEM; black dots indicate independent biological replicates, and grey and pink dots represent individual cells. (**e**) hTGFβ1–SiR-d12 surface binding to X. laevis receptor variants. One representative independent experiment is shown (n = 1; ~10 cells analysed); the experiment was repeated four times with similar results. Data are presented as mean ± SEM. No statistical testing was performed. Grey and pink dots represent individual cells. (**c**, **f**,) pSMAD2/3-sensitive $CAGA_{12}$ luciferase reporter activity of *C. carpio* (**c**) and *X. laevis* (**f**) receptor variants in the presence or absence of hTGFβ1 (0.2 nM), shown as fold induction (F.I.) of relative light units (RLU) relative to hTGFBR2 (+TGFβ1). Data are mean

± SD. n = 8 independent biological replicates (independent experiments) for (**c**) and n = 3 independent biological replicates for (**f**). Two-way ANOVA followed by Dunnett's multiple comparisons test (two-sided; vs LBD^{out} variants) was used. Exact P values: (**c**) cTGFBR2 vs cTGFBR2 LBD^{out}, p = 0.0002; cTGFBR2 LBD^{in} vs cTGFBR2 LBD^{out}, p < 0.0001; (**f**) xlTGFBR2 vs xlTGFBR2 LBD^{out}, p = 0.0011; xlTGFBR2 $LBD^{mid\text{-}in}$ vs xlTGFBR2 LBD^{out}, p < 0.0001; xlTGFBR2 LBD^{in} vs xlTGFBR2 LBD^{out}, p = 0.0027. Error bars represent SD. Source data are provided as a Source Data file. (**a, d**) Schematics created in BioRender. Trumpp, M. (2026), https://BioRender.com/8p64dhh.

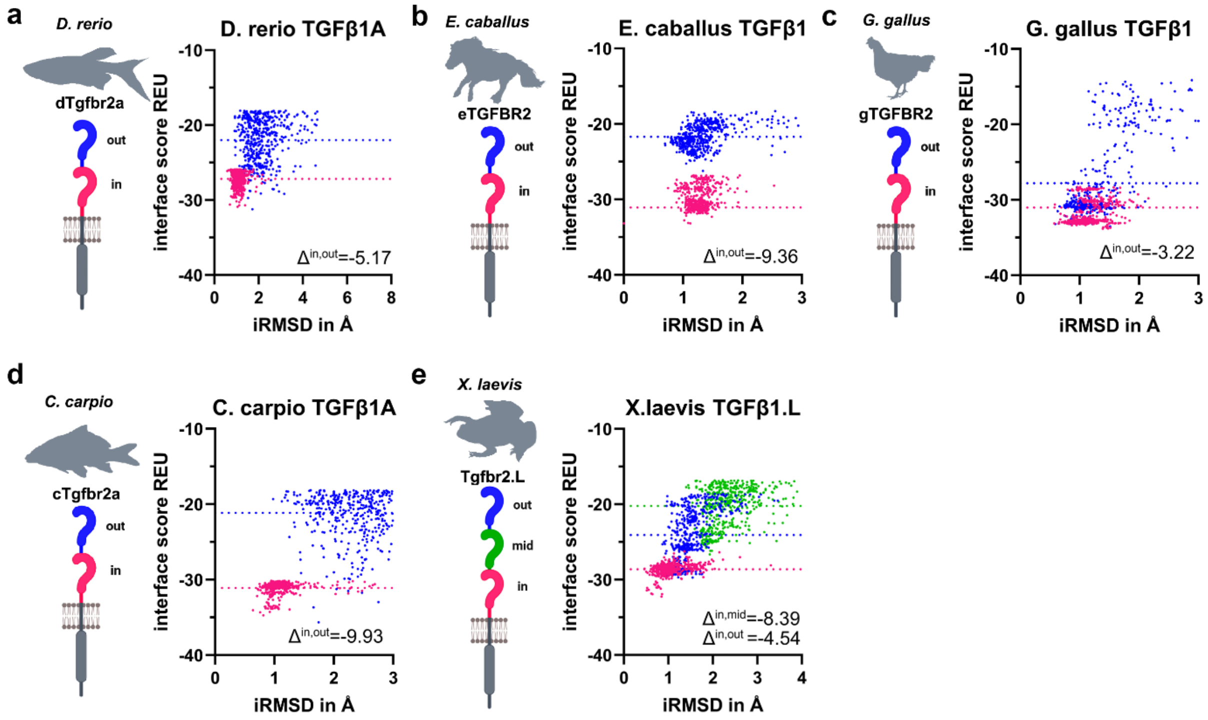

Supplementary Figure 10: *In silico* binding analysis of TGFBR2 orthologs with respective animal TGFβ1. *In silico* binding analysis via Rosetta docking and illustration of respective animal TGFBR2 LBD receptor domain structure as color reference of (**a**) *Danio rerio* (**b**) *Equus callabus* (**c**) *Gallus gallus* (**d**) *Cyprinus carpio* (**e**) *Xenopus laevis* TGFβ1 interactions with its respective single TGFBR2/Tgfbr2a/Tgfbr2.L domains, depicted as interface score (REU) in relation to interface root mean square deviation (iRMSD) in angstroms. REU differences of out and mid LBD mean are calculated against inner LBD mean value. Source data are provided as a Source Data file. (**a–e**) Schematics created in BioRender. Trumpp, M. (2026), https://BioRender.com/8p64dhh.

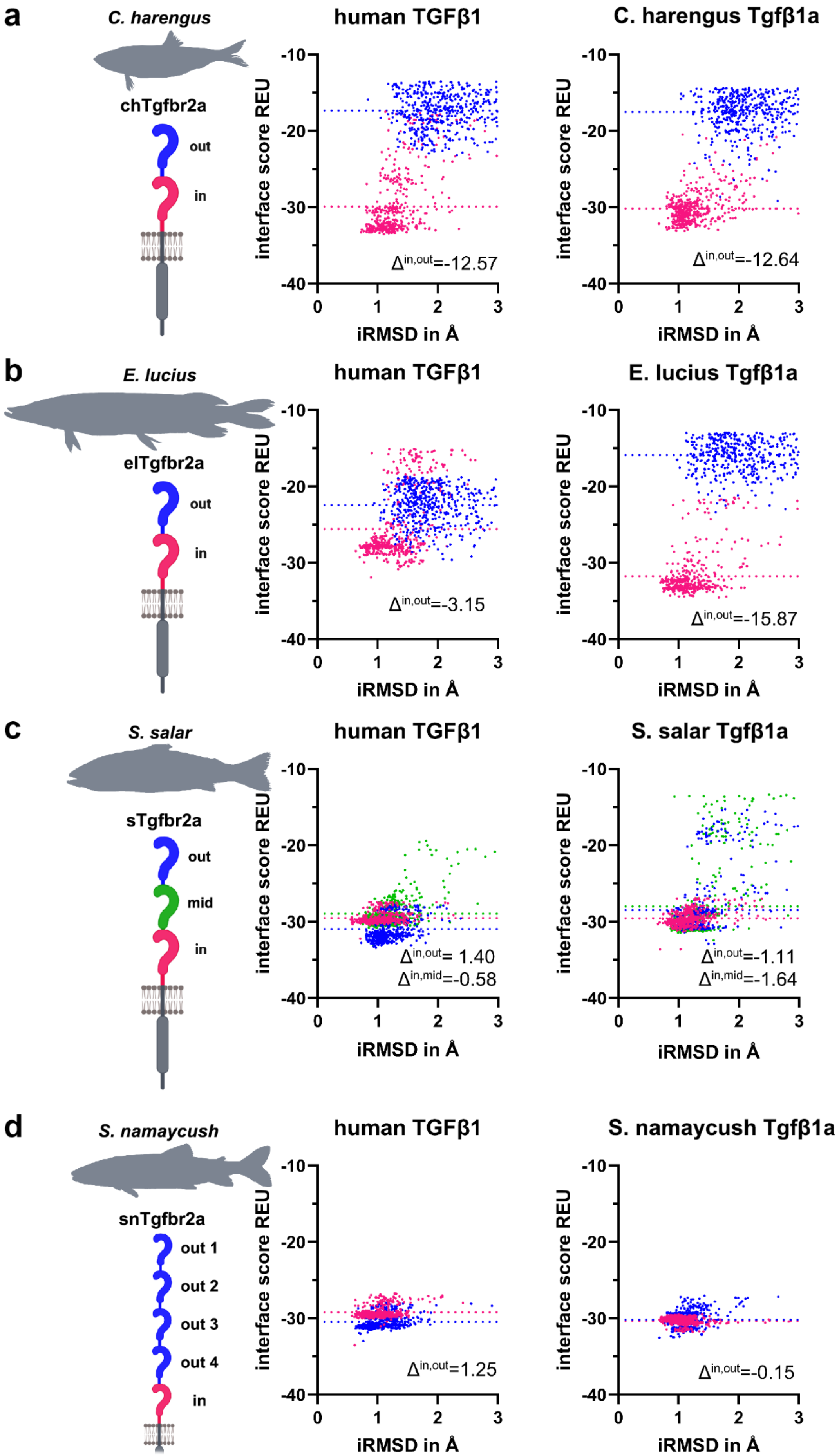

a
C. harengus
chTgfbr2a
out
in
human TGFβ1
C. harengus Tgfβ1a
interface score REU
iRMSD in Å
Δin,out=-12.57
Δin,out=-12.64
b
E. lucius
elTgfbr2a
out
in
human TGFβ1
E. lucius Tgfβ1a
Δin,out=-3.15
Δin,out=-15.87
c
S. salar
sTgfbr2a
out
mid
in
human TGFβ1
S. salar Tgfβ1a
Δin,out= 1.40
Δin,mid=-0.58
Δin,out=-1.11
Δin,mid=-1.64
d
S. namaycush
snTgfbr2a
out 1
out 2
out 3
out 4
in
human TGFβ1
S. namaycush Tgfβ1a
Δin,out=1.25
Δin,out=-0.15

Supplementary Figure 11: *In silico* binding analysis of ray-finned fish TGFBR2 orthologs with human and respective animal TGFβ1. Illustration of respective animal TGFBR2 LBD receptor domain structure as color reference and Rosetta docking via *in silico* binding analysis of (**a**) *Clupea harengus* (**b**) *Esox lucius* (**c**) *Salmo salar* (**d**) *Salvelinus namaycush* TGFβ1 *and* hTGFβ1 interactions with respective single Tgfbr2a domains, depicted as interface score (REU) in relation to interface root mean square deviation (iRMSD) in angstroms. REU differences of out and mid LBD mean are calculated against inner LBD mean value. Source data are provided as a Source Data file. (**a–d**) Schematics created in BioRender. Trumpp, M. (2026), https://BioRender.com/8p64dhh.

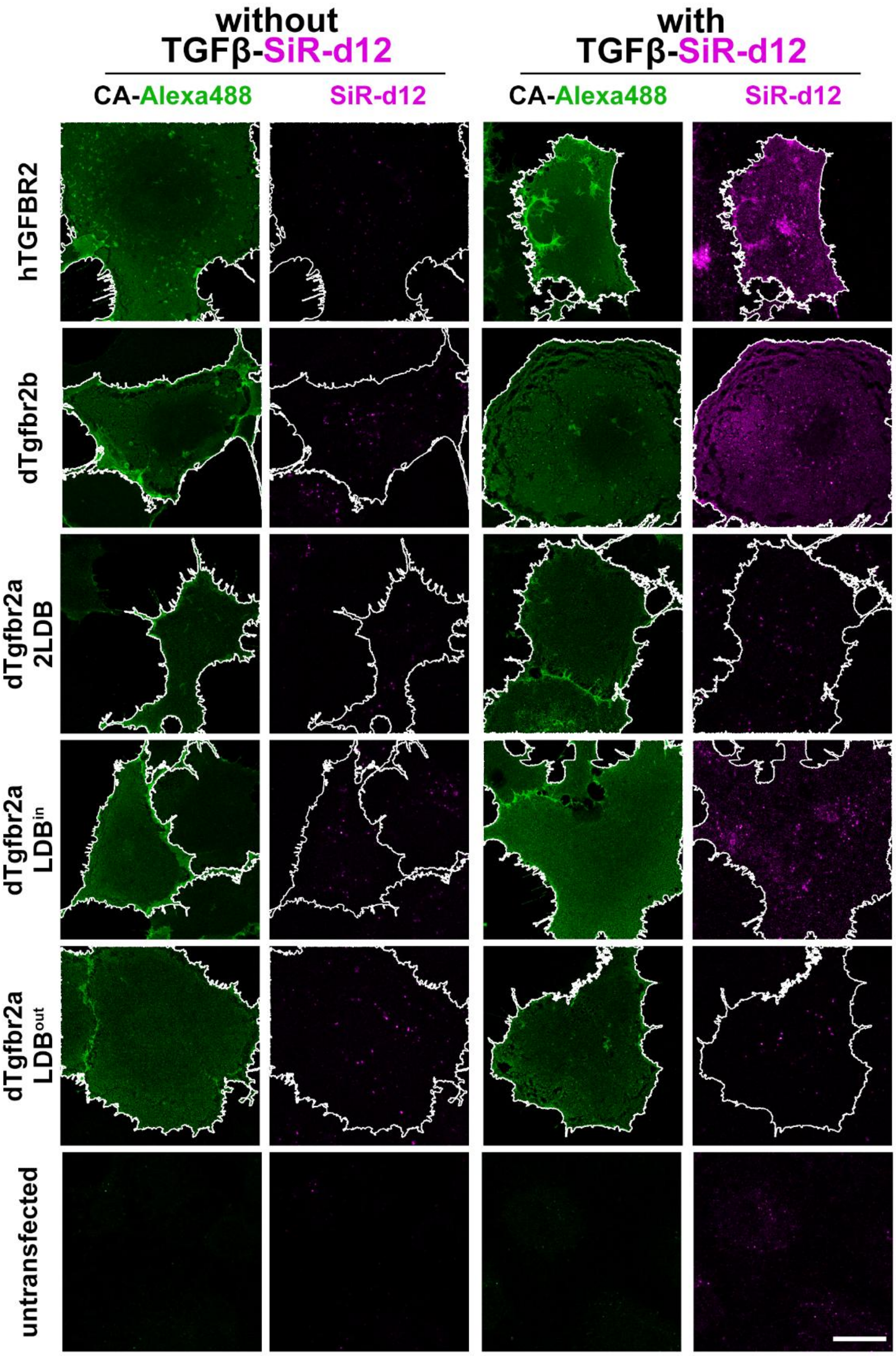

Supplementary Figure 12: Representative LSBA images of TGFβ1 bound to dTgfbr2a/b variants. Representative confocal microscopy images of LSBA for unstimulated and hTGFβ1-SiR-d12 stimulated COS-7 cells expressing human BMPR2 or *danio rerio* variants *(*dTgfbr2b, dTgfbr2a (2LBD), dTgfbr2a-LBD^{in}, dTgfbr2a-LBD^{out}) or untransfected control. Scale bar ≙ 20 µm.

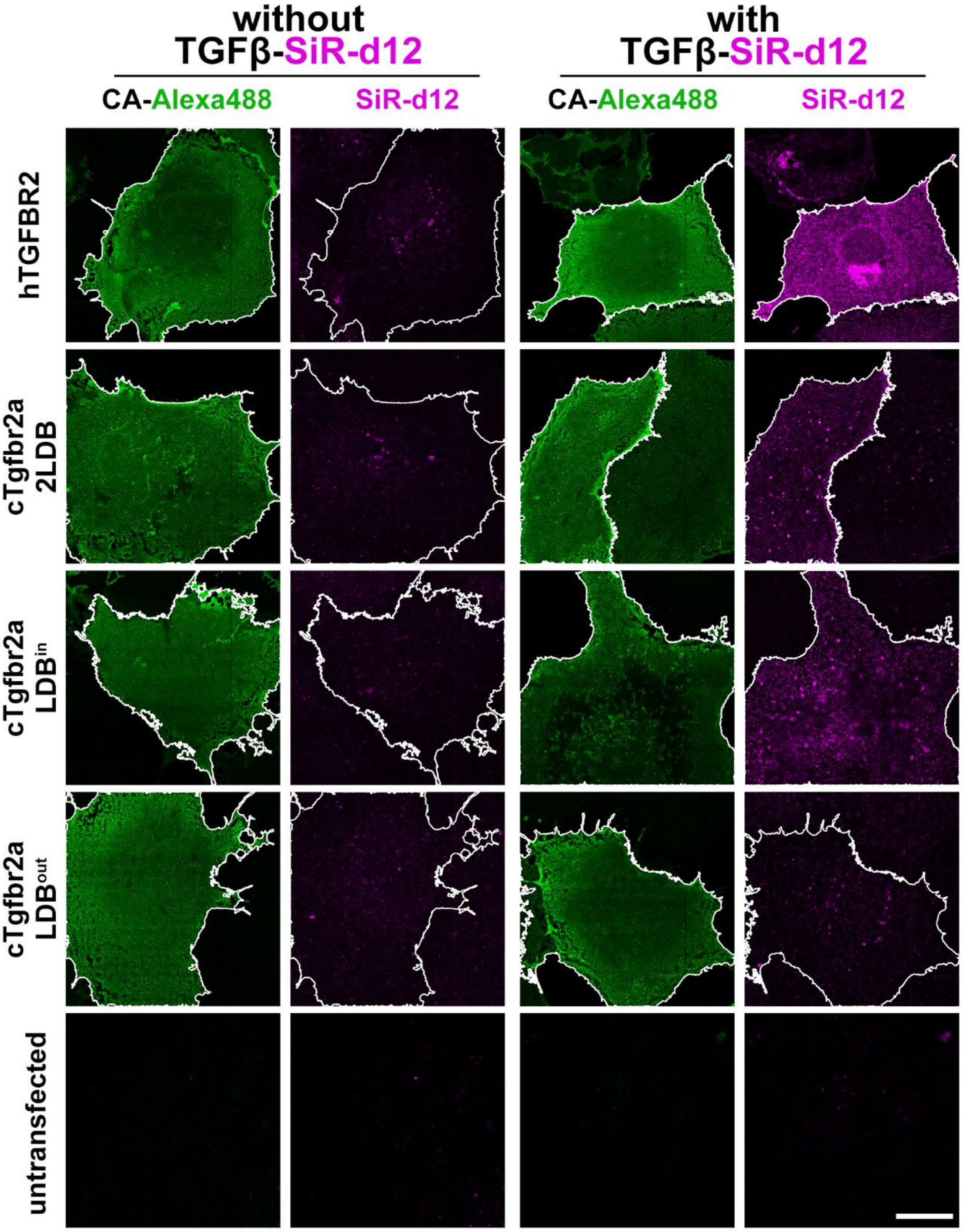

Supplementary Figure 13: Representative LSBA images of TGFβ1 bound to cTgfbr2a variants. Representative confocal microscopy images of LSBA for unstimulated and hTGFβ1-SiR-d12 stimulated COS-7 cells expressing human TGFBR2 or *Cyprinus carpio* variants (cTgfbr2a (2LBD), cTgfbr2a-LBD^{in}, cTgfbr2a-LBD^{out}) or untransfected control. Scale bar ≙ 20 µm.

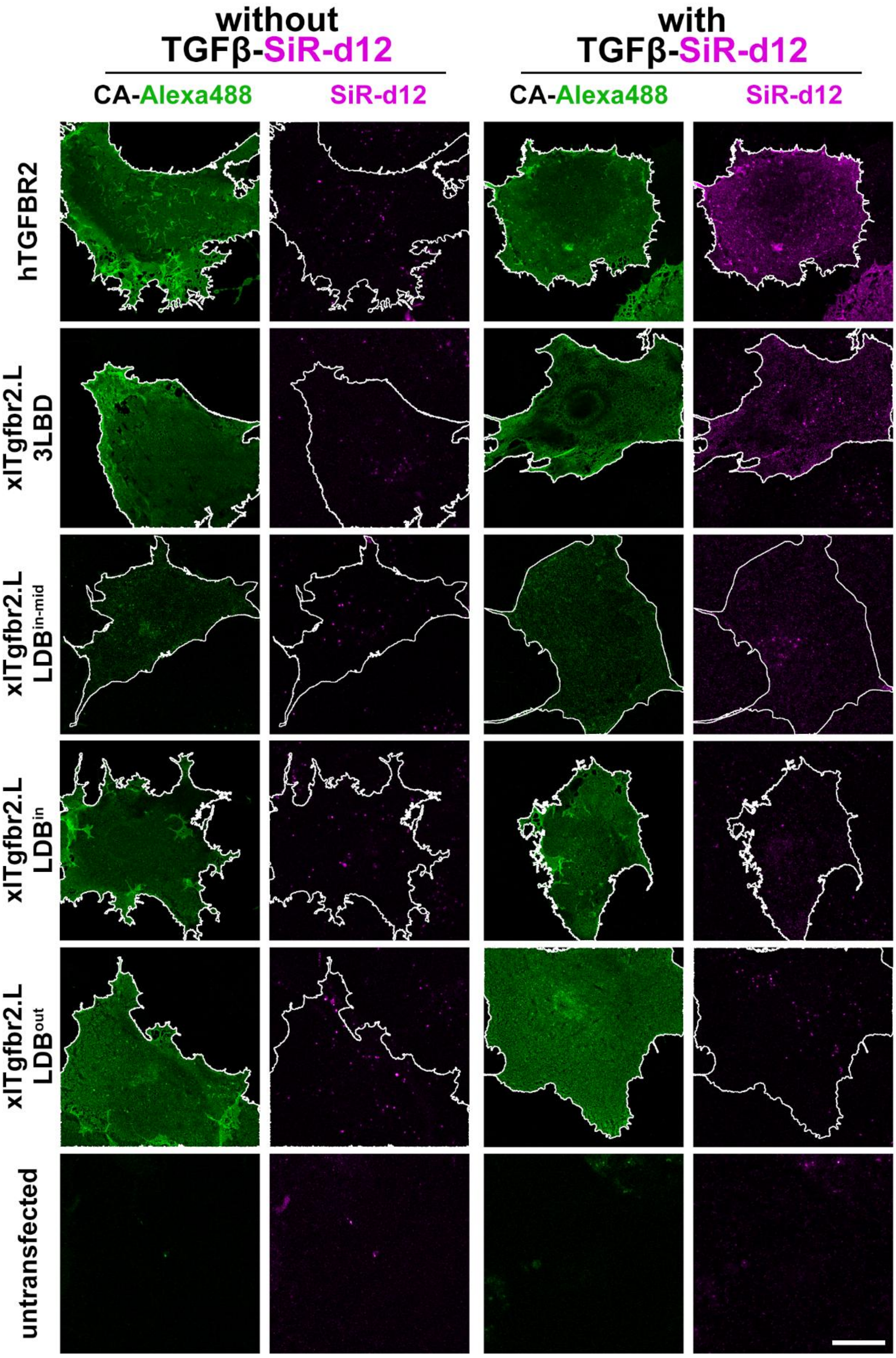

Supplementary Figure 14: Representative LSBA images of TGFβ1 bound to xlTgfbr2.L variants. Representative confocal microscopy images of LSBA for unstimulated and hTGFβ1-SiR-d12 stimulated COS-7 cells expressing human TGFBR2 or *Xenopus laevis* variants (xlTgfbr2.L (3LBD), xlTgfbr2.L-LBD^{mid-in}, xlTgfbr2.L-LBD^{in}, xlTgfbr2.L-LBD^{out}) or untransfected control. Scale bar ≙ 20 µm.

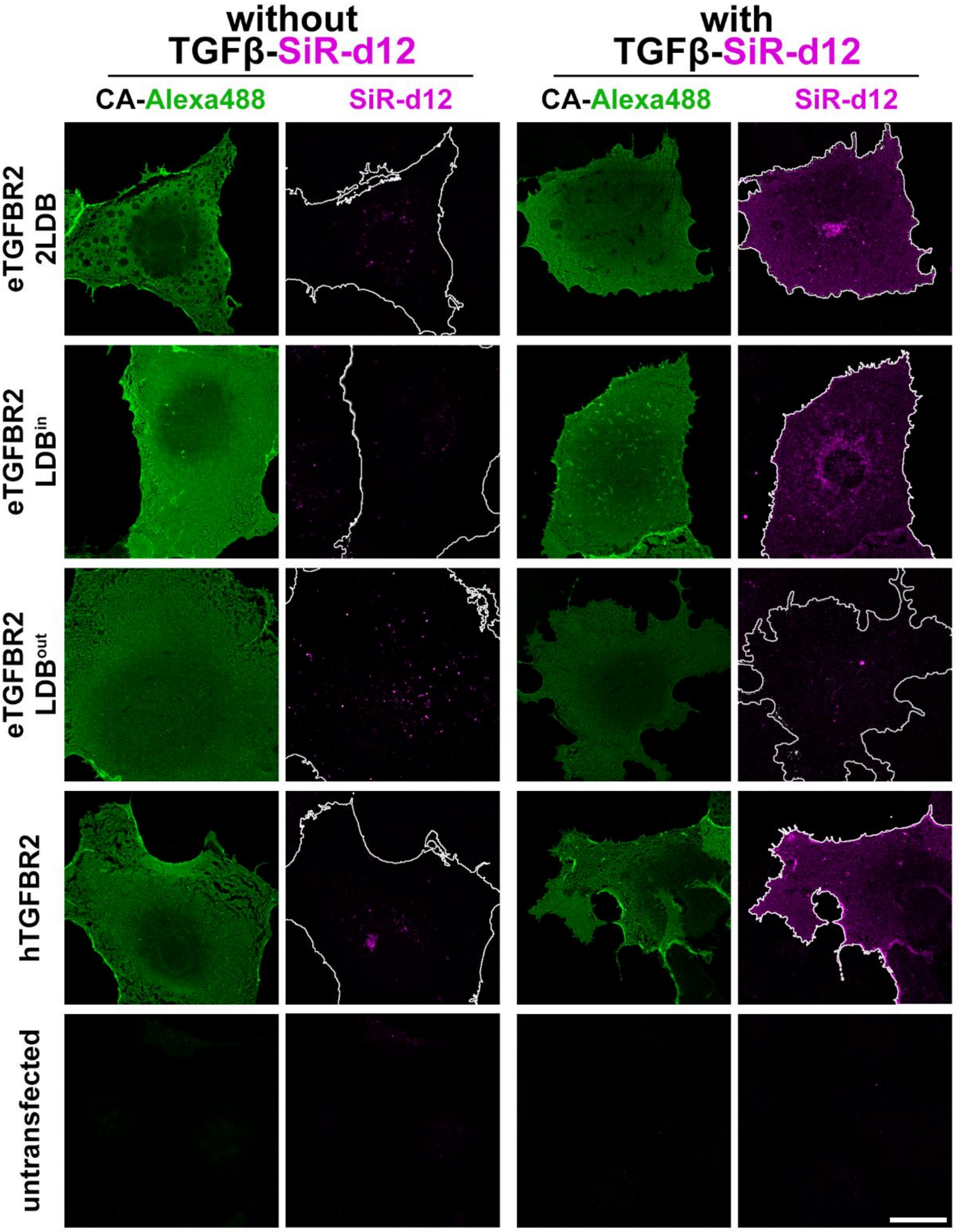

Supplementary Figure 15: Representative LSBA images of TGFβ1 bound to eTGFBR2 variants. Representative confocal microscopy images of LSBA for unstimulated and hTGFβ1-SiR-d12 stimulated COS-7 cells expressing *Equus caballus* variants *(*eTGFBR2 (2LBD), eTGFBR2-LBD^{in}, eTGFBR2-LBD^{out}) and human TGFBR2 or untransfected control. Scale bar ≙ 20 µm.

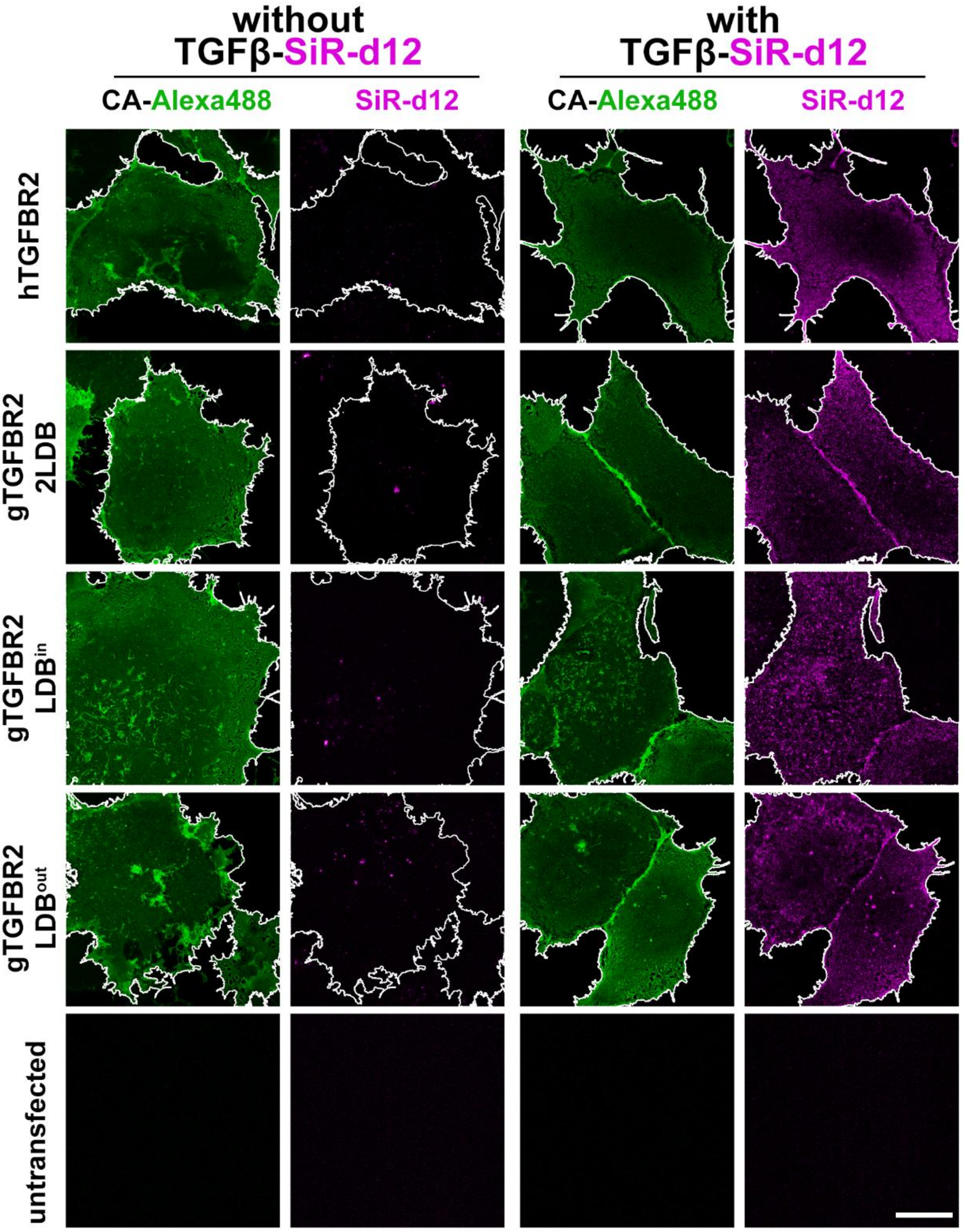

Supplementary Figure 16: Representative LSBA images of TGFβ1 bound to gTGFBR2 variants. Representative confocal microscopy images of LSBA for unstimulated and hTGFβ1-SiR-d12 stimulated COS-7 cells expressing human TGFBR2 and *Gallus gallus* variants *(*gTGFBR2 (2LBD), gTGFBR2-LBD^{in}, gTGFBR2-LBD^{out}) and human TGFBR2 or untransfected control. Scale bar ≙ 20 µm.

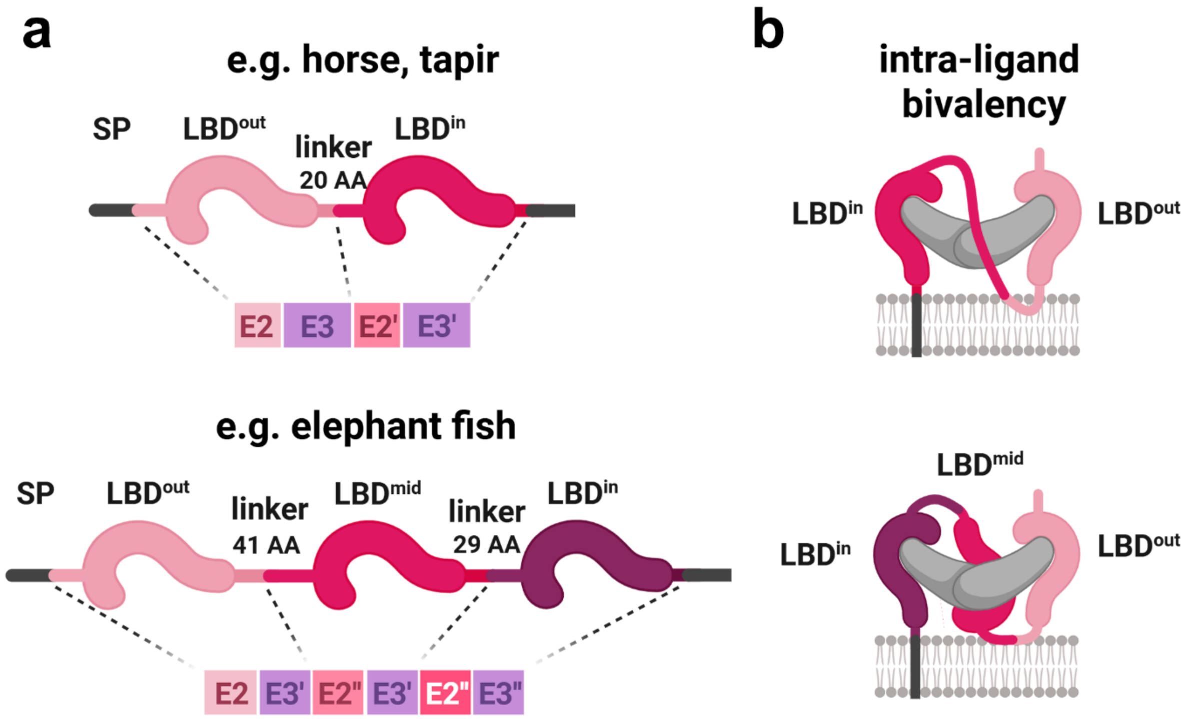

c

Species	Protein	Linker Type	Linker Length
Homo sapiens	hTGFBR2 2LBD	LBD^{out}-LBD^{in}	20 AA
Equus caballus	TGFBR2	LBD^{out}-LBD^{in}	20 AA
Tapirus indicus	TGFBR2	LBD^{out}-LBD^{in}	20 AA
Gallus gallus	TGFBR2	LBD^{out}-LBD^{in}	13 AA
Xenopus laevis	Tgfbr2.L	LBD^{out}-LBD^{mid}	33 AA
Xenopus laevis	Tgfbr2.L	LBD^{mid}-LBD^{in}	13 AA
Salmo salar	Tgfbr2a	LBD^{out}-LBD^{in}	13 AA
Esox lucius	Tgfbr2a	LBD^{out}-LBD^{in}	12 AA
Clupea harengus	Tgfbr2a	LBD^{out}-LBD^{in}	44 AA
Salvelinus namaycush	Tgfbr2a	LBD^{out}-LBD^{in}	13 AA
Danio rerio	Tgfbr2a	LBD^{out}-LBD^{in}	21 AA
Cyprinus carpio	Tgfbr2a	LBD^{out}-LBD^{in}	24 AA
Paramormyrops kingsleyae	Bmpr2a	LBD^{mid}-LBD^{in}	29 AA
Paramormyrops kingsleyae	Bmpr2a	LBD^{out}-LBD^{mid}	45 AA
Gnathonemus petersii	Bmpr2a	LBD^{mid}-LBD^{in}	29 AA
Gnathonemus petersii	Bmpr2a	LBD^{out}-LBD^{mid}	41 AA
Erpetoichthys calabaricus	BMPR2	LBD^{mid}-LBD^{in}	30 AA
Erpetoichthys calabaricus	BMPR2	LBD^{out}-LBD^{mid}	30 AA

Supplementary Figure 17: Linker architecture in TGFBR2 and BMPR2 variants. (**a**) Schematic illustration of exon architecture of receptors with duplicated (i.e. TGFBR2) or triplicated (i.e. BMPR2) extracellular LBDs. Linker length is defined by the residues encoded at the junction of exons E3 and E2′. (**b**) Illustration of two potential modes of intra-ligand bivalency of receptors with two or three LBDs. Intra-ligand bivalency may require a sufficiently

long and flexible linker between the LBDs. The presence of a third LBD could further affect the likelihood of bivalent binding. (**c**) Cross-species overview of linker length variation in duplicated LBD TGFBR2 and BMPR2 variants. (**a, b**) Schematics created in BioRender. Trumpp, M. (2026), https://BioRender.com/8p64dhh.

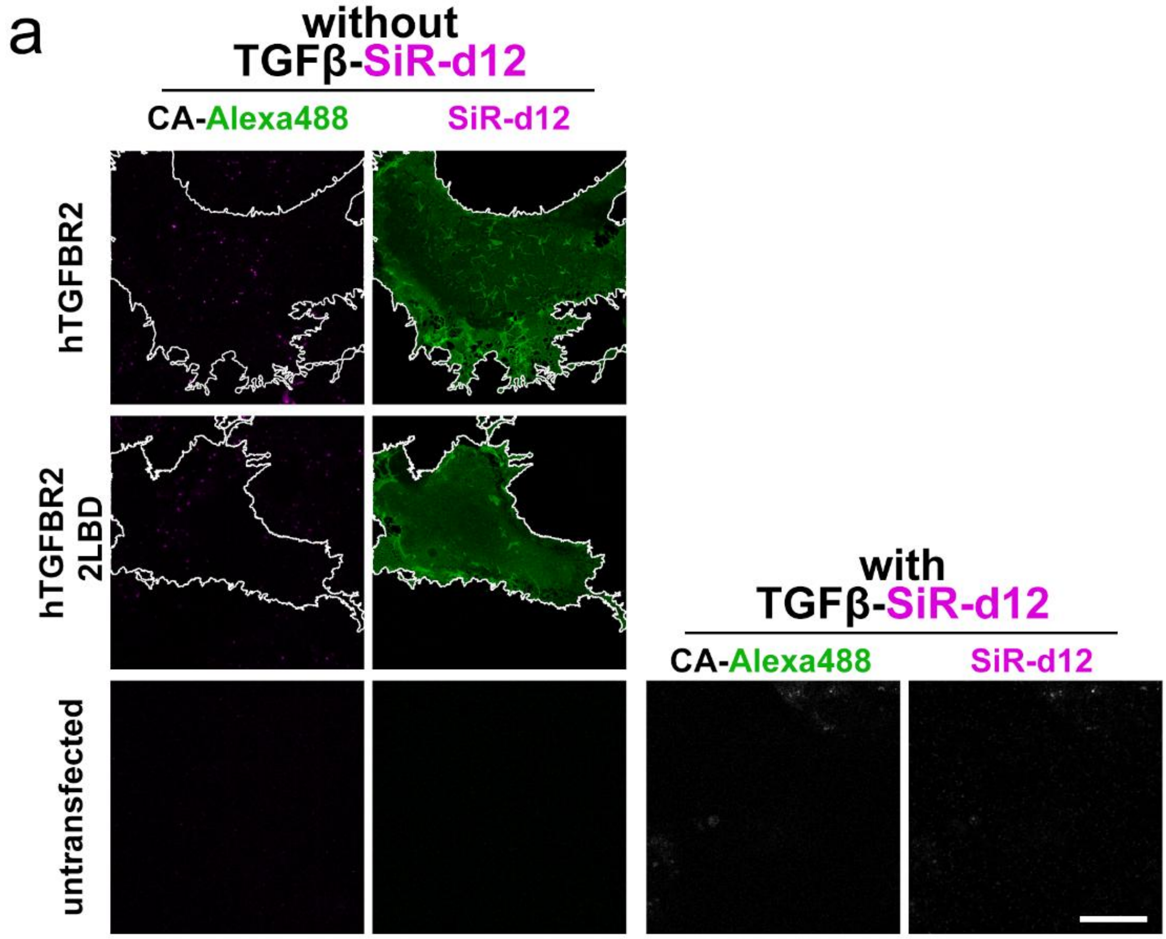

Supplementary Figure 18: Representative LSBA images of TGFβ1 bound to hTGFBR2 variants. Representative confocal microscopy images of LSBA for unstimulated COS-7 cells expressing hTGFBR2 or hTGFBR2-2LBD or untransfected control and hTGFβ1-SiR-d12 stimulated untransfected cells. Scale bar ≙ 20 μm.

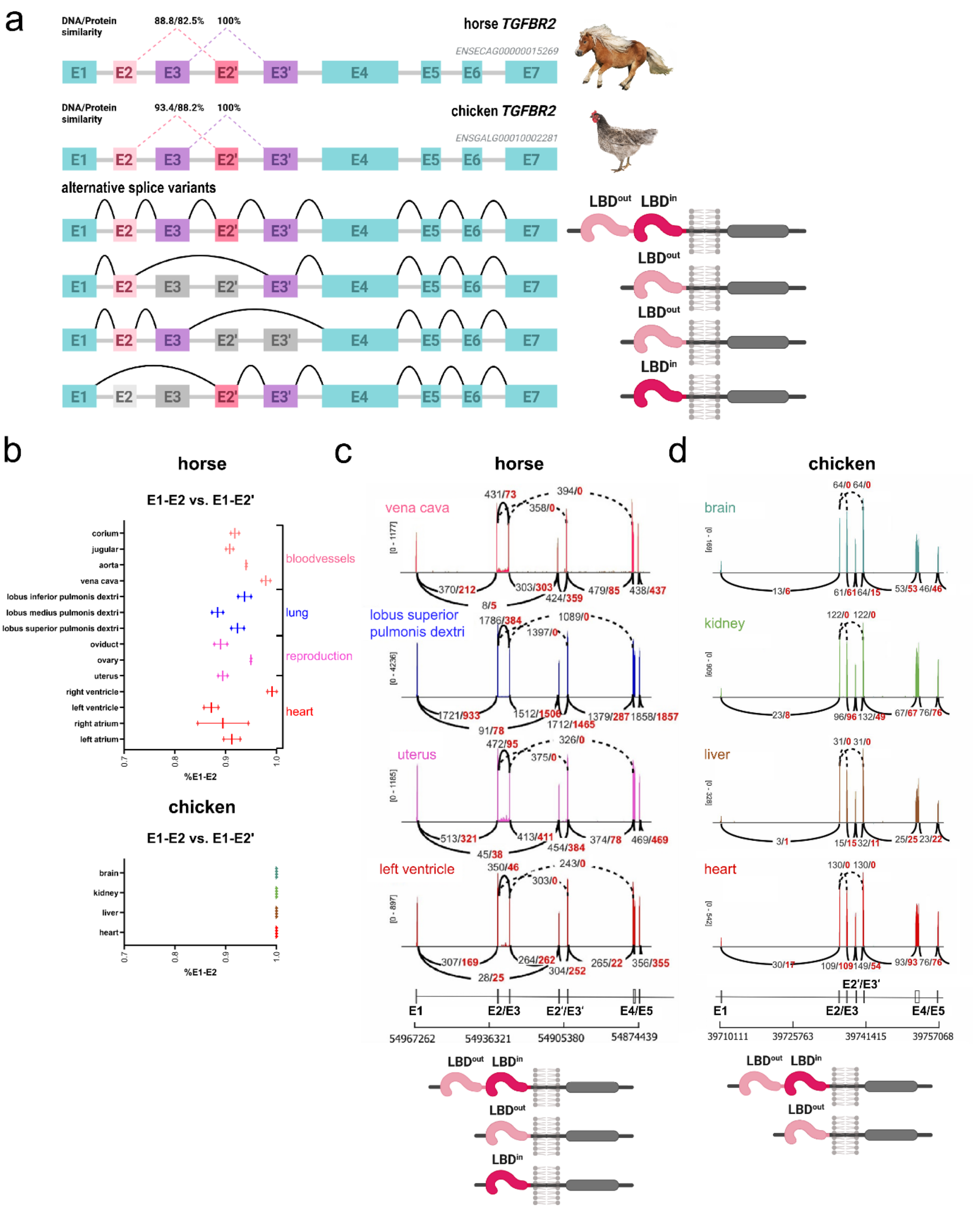

Supplementary Figure 19: Alternative splice variants of TGFBR2 in horse and chicken. (**a, top**) Exon organization of horse eTGFBR2 and chicken gTGFBR2 with DNA/protein similarity percentages between corresponding exons. (**bottom**) Illustration of alternative splice variants, showing different combinations of exons leading to 2LBD, LBD_{out}, LBD_{in} receptor variants. (**b**) Summary of relative usage 2a/2b usage across 14 horse tissues. Tissues are indicated on the left (reproduction, blood vessels, lung, heart). Tissues used for Sashimi plots are highlighted. (**c,d**) Sashimi plot of the E1 to E5 region of horse (left) and chicken (right) TGFBR2. Exon coverage is shown on the left. Junctions are indicated by lines, junctions not supported by unique reads are shown as dotted lines. Next to each junction the black number

shows all reads mapped to this junction, the red number reports unique reads only. Source data are provided as a Source Data file. (**a, c–d**) Schematics created in BioRender. Trumpp, M. (2026), https://BioRender.com/8p64dhh.

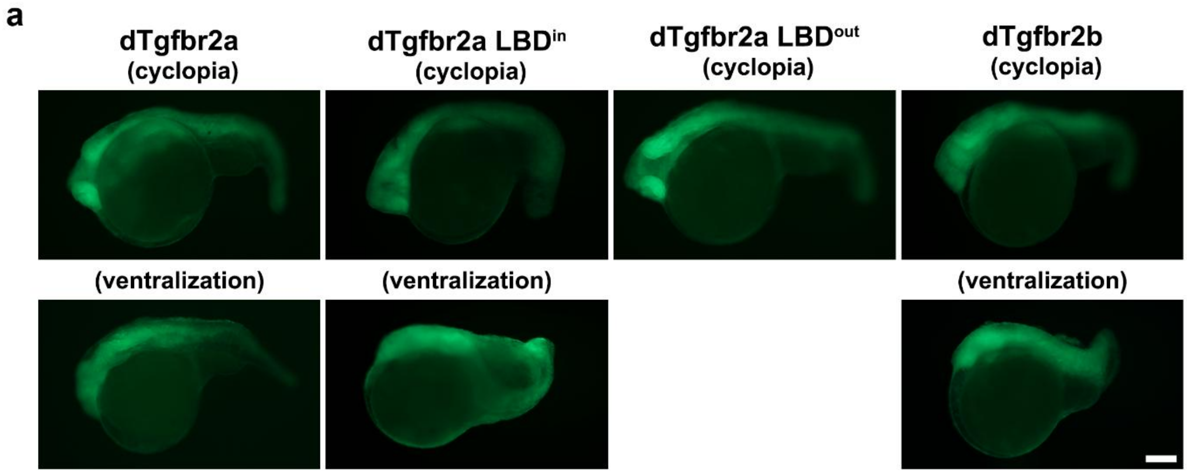

Supplementary Figure 20: Coinjected membrane bound PMT-mEGFP expression control in zebrafish embryos. (**a**) Expression of coinjected PMT-mEGFP allows selection of dTgfbr2 overexpression embryos. Scale bar = 100 µm.

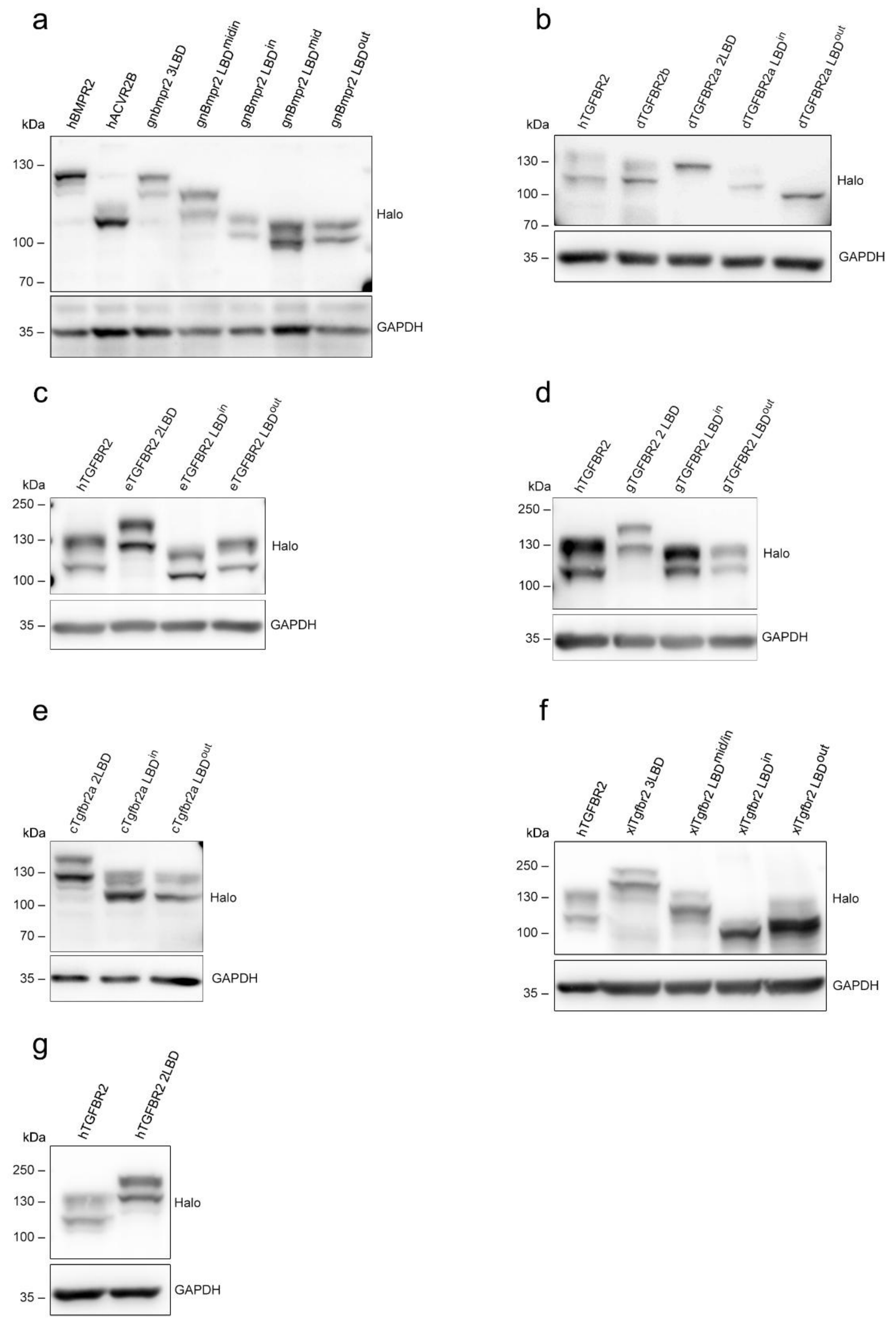

Supplementary Figure 21: Western blot expression controls of Halo-tagged receptor constructs. Western blot analysis of HEK293T cell transiently expressing (**a**) *Gnathonemus petersii* receptor variants *(*gBmpr2a (3LBD), gBmpr2a-LBD^{midin}, gBmpr2a-LBD^{in}, gBmpr2a-LBD^{mid}, gBmpr2a-LBD^{out}) or hBMPR2 and hACVR2B as size reference (**b**) *Danio rerio* receptor variants *(*dTgfbr2b, dTgfbr2a (2LBD), dTgfbr2a-LBD^{in}, dTgfbr2a-LBD^{out}) or hTGFBR2 as size reference (**c**) *Equus caballus* variants *(*eTGFBR2 (2LBD), eTGFBR2-LBD^{in}, eTGFBR2-LBD^{out}) and hTGFBR2 as size reference (**d**) *Gallus gallus* variants *(*gTGFBR2 (2LBD), gTGFBR2-LBD^{in}, gTGFBR2-LBD^{out}) and hTGFBR2 as size reference (**e**) *Cyprinus carpio* variants *(*cTgfbr2a (2LBD), cTgfbr2a-LBD^{in}, cTgfbr2a-LBD^{out}) or hTGFBR2 as size reference (**f**)

Xenopus laevis variants (xlTgfbr2.L (3LBD), xlTgfbr2.L-LBD^{mid-in}, xlTgfbr2.L-LBD^{in}, xlTgfbr2.L-LBD^{out}) or hTGFBR2 as size reference (**g**) hTGFBR2 and hTGFBR2-2LBD. GAPDH was used as loading reference.

Supplementary Table 1: N-terminally tagged HaloTag expression constructs

Plasmid Name	Description	Backbone	Resistance
eTGFBR2-Halo	full length eTGFBR2 with N-terminal HaloTag	pcDNA3.1	Ampicillin
eTGFBR2 LBD^{in}-Halo	eTGFBR2 LBD^{in} variant with N-terminal HaloTag	pcDNA3.1	Ampicillin
eTGFBR2 LBD^{out}-Halo	eTGFBR2 LBD^{out} variant with N-terminal HaloTag	pcDNA3.1	Ampicillin
dTgfbr2a-Halo	full length dTgfbr2a with N-terminal HaloTag	pcDNA3.1	Ampicillin
dTgfbr2a LBD^{in}-Halo	dTgfbr2a LBD^{in} variant with N-terminal HaloTag	pcDNA3.1	Ampicillin
dTgfbr2a LBD^{out}-Halo	dTgfbr2a LBD^{out} variant with N-terminal HaloTag	pcDNA3.1	Ampicillin
dTgfbr2b-Halo	dTgfbr2b with N-terminal HaloTag	pcDNA3.1	Ampicillin
cTgfbr2a-Halo	full length cTgfbr2a with N-terminal HaloTag	pcDNA3.1	Ampicillin
cTgfbr2a LBD^{in}-Halo	cTgfbr2a LBD^{in} variant with N-terminal HaloTag	pcDNA3.1	Ampicillin
cTgfbr2a LBD^{out}-Halo	cTgfbr2a LBD^{out} variant with N-terminal HaloTag	pcDNA3.1	Ampicillin
gnBmpr2a-Halo	full length gnBmpr2a with N-terminal HaloTag	pcDNA3.1	Ampicillin
gnBmpr2a LBD^{in}-Halo	gnBmpr2a LBD^{in} variant with N-terminal HaloTag	pcDNA3.1	Ampicillin
gnBmpr2a LBD^{mid-in}-Halo	gnBmpr2a LBD^{mid-in} variant with N-terminal HaloTag	pcDNA3.1	Ampicillin
gnBmpr2a LBD^{out}-Halo	gnBmpr2a LBD^{out} variant with N-terminal HaloTag	pcDNA3.1	Ampicillin
gnBmpr2a LBD^{mid}-Halo	gnBmpr2a LBD^{mid} variant with N-terminal HaloTag	pcDNA3.1	Ampicillin
gTGFBR2-Halo	full length gTGFBR2 with N-terminal HaloTag	pcDNA3.1	Ampicillin
gTGFBR2 LBD^{in}-Halo	gTGFBR2 LBD^{in}-Halo variant with N-terminal HaloTag	pcDNA3.1	Ampicillin
gTGFBR2 LBD^{out}-Halo	gTGFBR2 LBD^{in}-Halo variant with N-terminal HaloTag	pcDNA3.1	Ampicillin
xlTgbfr2.L – blunt end	full length xlTgbfr2.L in blunt end vector	pCR™-Blunt-vector	Ampicillin
xlTgbfr2.L	full length xlTgbfr2.L with N-terminal HaloTag	pcDNA3.1	Ampicillin
xlTgbfr2.L LBD^{in}	xlTgbfr2.L LBD^{in} variant with N-terminal HaloTag	pcDNA3.1	Ampicillin
xlTgbfr2.L LBD^{mid-in}	xlTgbfr2.L LBD^{mid-in} variant with N-terminal HaloTag	pcDNA3.1	Ampicillin
pcs2+PMT-mEGFP	Membrane bound mEGFP: Gift from Dr. Thorsten Wohland (Balasubramanian et. al, 2022, RRID: Addgene_203777), subcloned into pCS2+ backbone for capped mRNA synthesis (Le et al., 2025)	pcs2+	Ampicillin
xlTgbfr2.L LBD^{out}	xlTgbfr2.L LBD^{out} variant with N-terminal	pcDNA3.1	Ampicillin

Supplementary Table 2: Oligonucleotides used for molecular cloning of LBD-Halo constructs

Name	Oligonucleotide-sequence (5'-3')	
	for	rev
eTGFBR2	CATAATTTTCAGTGAAAACCCCGACTTG<br>CTTTTAGTTATCTTTCAAG	ATAACTAAAAGCAAGTCGGGGTTTTCACTGA<br>AAATTATGTGGTCGTTGC
eTGFBR2<br>LBD^{in}	GCACTGCGGCTGCTGGATCCGCAGAAA<br>TCGGTACTGGCTTTCCATTC	ACTAAAAGCAAGTCGGGGTTTTCACTGAAAA<br>TTATGTGGTCG
eTGFBR2<br>LBD^{out}	AACCCCGACTTGCTTTTAGTTATCTTTC<br>AAGTGACAGGGG	GGATCCAGCAGCCGCAGTGCTGACCAGCA<br>GGATGGTCCATGG
dTgfbr2a	GGTGGAGAATTCCGCATCTGCAGCTCC<br>TGTAAGCCTCATCCAGTCAACTG	GCTCGAGCGGCCGCCTACATCTCTTGTATC<br>CCAGAATCC
dTgfbr2a<br>LBD^{in}	GGTGGAGAATTCTTGTCGAAGACAAACT<br>CCAGCAGC	GCTCGAGCGGCCGCCTACATCTCTTGTATC<br>CCAGAATCC
dTgfbr2a<br>LBD^{out}	GATTTGAAACAATATCCAGGGGTTCAG	CAATTTCTCCTCCTCAGTGAGTTTG
dTgfbr2b	GGTGGAGAATTCCGCGGAGTCGATGGA<br>AATAATCC	GCTCGAGCGGCCGCTCATTTGTCGTCGCTC<br>ACGCTCACG
cTgfbr2a	GGTGGAGAATTCAATCCACACAGTGTG<br>TGTAAGTCATGTGACC	GCTCGAGCGGCCGCCTATTTCTCATCATTC<br>CCAGAATCC
cTgfbr2a<br>LBD^{in}	GATAACGACACCAAGTTAGTTATATTG	ATTGAATTCTCCACCTCCACCTCCGC
cTgfbr2a<br>LBD^{out}	GTTTCGTCTCCTCCAGGAGACTATAAG	GAGTCTGAACCCTCCTTTCGTTTC
gnBmpr2a	GGTGGAGAATTCGCCCAAGCGGAAGAC<br>CGCGTGTGC	GCTCGAGCGGCCGCCTAATTAACGGTAGGT<br>GACACG
gnBmpr2a^{in}	GGTGGAGAATTCGCACCTAATGAAGAA<br>AGGGAATGC	GCTCGAGCGGCCGCCTAATTAACGGTAGGT<br>GACACG
gnBmpr2a<br>$^{mid-in}$	GGTGGAGAATTCGCCGAAAGAGACGAA<br>TTGC	GCTCGAGCGGCCGCCTAATTAACGGTAGGT<br>GACACG
gnBmpr2a^{out}<br>(Gibson)	**Backbone:**<br>CCGCTGTTCGGTCCAACATCCACTCAA<br>CCCCTCTATCCAC<br>**LBD^{out}:**<br>GAGGTGGAGGTGGAGAATTCGCCCAAG<br>CGGAAGACCGCGTG	**Backbone:**<br>GAATTCTCCACCTCCACCTCCGCCGGAAAT<br>CTCGAGCGTCG<br>**LBD^{out}:**<br>GATGTTGGACCGAACAGCGGAAAATCTTCG<br>GTGAAATTAAGATTAC
gnBmpr2a^{mid}<br>(Gibson)	**Backbone:**<br>CCGCTGTTCGGTCCAACATCCACTCAA<br>CCCCTCTATCCAC<br>**LBD^{mid}:**<br>GAGGTGGAGGTGGAGAATTCGCCGAAA<br>GAGACGAATTGCAATGC	**Backbone:**<br>GAATTCTCCACCTCCACCTCCGCCGGAAAT<br>CTCGAGCGTCG<br>**LBD^{mid}:**<br>GATGTTGGACCGAACAGCGGAGACAGATCG<br>GTGAAGTTTAC
gTGFBR2 &<br>gTGFBR2<br>LBD^{out}	GGTGGAGAATTCAGAAGCAAGGAAAAT<br>GGACTGC	GCTCGAGCGGCCGCCTACTTGGCGGTGGT<br>CACGGAGC
gTGFBR2,<br>gTGFBR2-<br>LBD^{out}<br>(Gibson)	**Backbone:**<br>GGCATCCAGATGGTGTGCGAGACCCTT<br>ATCGAGTGCTGGG<br>**Exon 6/7 Del:**<br>TGCCCATTGAACTGGACATTGTTGTTGG<br>CAAAGGAAGGTTTGCTGAAGTG	**Backbone:**<br>GGCATCCAGATGGTGTGCGAGACCCTTATC<br>GAGTGCTGGG<br>**Exon 6/7 Del:**<br>TCGCACACCATCTGGATGCCCTGATGGTTA<br>AGCCAGGAGCTGG
gTGFBR2<br>LBD^{in}	CCCCATAAGCCAGAGGAGAAAGATGAA<br>ATTTCC	CCTAAATCTGTTTGCAGAAAAAATAAG
xlTgbfr2.L –<br>blunt end	GGTACCTCCACCAAGGGCGAATTCTGC	ACATTTCCCTTATACCTGGGTTCTAGTC
xlTgbfr2.L<br>LBD^{in}<br>(Gibson)	**Backbone:**<br>GATGACCCATCAAAAGACATACTTGTGA<br>AAGCTCTTCTCATC<br>**LBD^{in}:**<br>GAGGTGGAGGTGGAGAATTCAGTATAA<br>AGGATTGTGC	**Backbone:**<br>GAATTCTCCACCTCCACCTCCGCCGGAAAT<br>CTCGAGCGTCGACAGCC<br>**LBD^{in}:**<br>ATGTCTTTTGATGGGTCATCTTTAGAAAAAA<br>ACAAGTTTTCATTACACAGC
xlTgbfr2.L<br>LBD^{mid-in}<br>(Gibson)	**Backbone:**<br>GATGACCCATCAAAAGACATACTTGTGA<br>AAGCTCTTCTCATC	**Backbone:**<br>GAATTCTCCACCTCCACCTCCGCCGGAAAT<br>CTCGAGCGTCGACAGCC

	**LBD$^{mid\text{-}in}$:**<br>CCGGCGGAGGTGGAGGTGGAGAATTC CCTGTTCATGTACCTGTTGTATGC	**LBD$^{mid\text{-}in}$:**<br>ATGTCTTTTGATGGGTCATCTTTAGAAAAAA ACAAGTTTTCATTACACAGC
xlTgbfr2.L LBD^{out} (Gibson)	**Backbone:**<br>GATGACCCATCAAAAGACATACTTGTGA AAGCTCTTCTCATC<br>**LBD^{out}:**<br>GAGGTGGAGGTGGAGAATTCTACCTGG GTTCTAGTCGTTTATGC	**Backbone:**<br>GAATTCTCCACCTCCACCTCCGCCGGAAAT CTCGAGCGTCGACAGCC<br>**LBD^{out}:**<br>ATGCTTTTGATGGGTCATCATCTGTGAAGAC TATTTGTTCATTGC

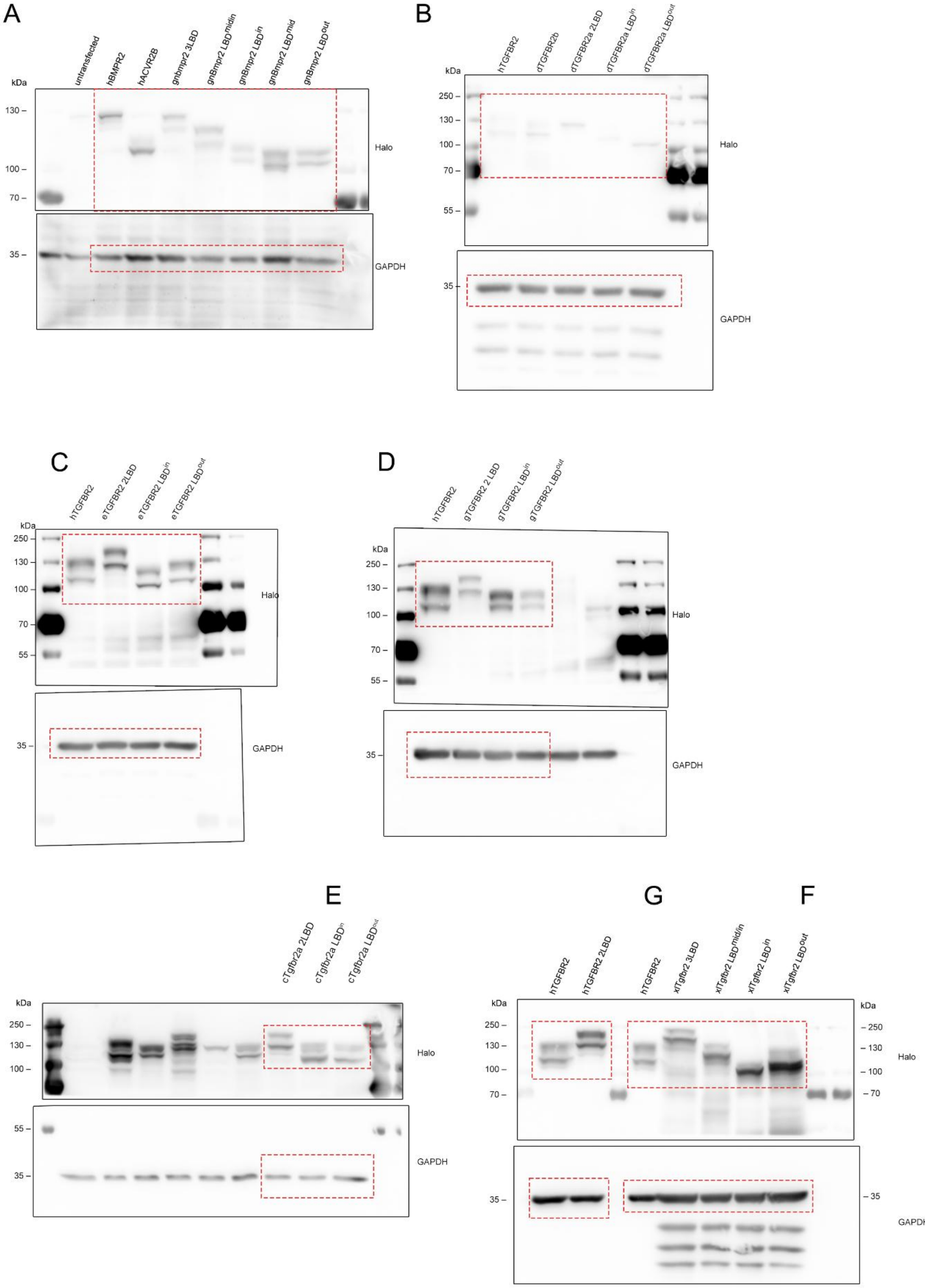

Supplementary Figure 22: (A-G) Uncropped Western blots
