# Supplementary material for: Recurrent evolution of ligand-binding domain multiplicity fine-tunes TGFβ signaling in vertebrates
Source: Nat Commun. 2026 May 19;17:4458. doi: 10.1038/s41467-026-73340-5 (PMC13187141; doi:10.1038/s41467-026-73340-5)
Supplement: Supplementary file 3 — Reporting Summary [file 41467_2026_73340_MOESM3_ESM.pdf]

nature portfolio

Corresponding author(s): Jerome Jatzlau, Petra Knaus

Last updated by author(s): Apr 28, 2026

Reporting Summary

Nature Portfolio wishes to improve the reproducibility of the work that we publish. This form provides structure for consistency and transparency in reporting. For further information on Nature Portfolio policies, see our Editorial Policies and the Editorial Policy Checklist.

Statistics

For all statistical analyses, confirm that the following items are present in the figure legend, table legend, main text, or Methods section.

n/a	Confirmed	
☐	☒	The exact sample size (*n*) for each experimental group/condition, given as a discrete number and unit of measurement
☐	☒	A statement on whether measurements were taken from distinct samples or whether the same sample was measured repeatedly
☐	☒	The statistical test(s) used AND whether they are one- or two-sided<br>*Only common tests should be described solely by name; describe more complex techniques in the Methods section.*
☒	☐	A description of all covariates tested
☐	☒	A description of any assumptions or corrections, such as tests of normality and adjustment for multiple comparisons
☐	☒	A full description of the statistical parameters including central tendency (e.g. means) or other basic estimates (e.g. regression coefficient) AND variation (e.g. standard deviation) or associated estimates of uncertainty (e.g. confidence intervals)
☐	☒	For null hypothesis testing, the test statistic (e.g. *F*, *t*, *r*) with confidence intervals, effect sizes, degrees of freedom and *P* value noted<br>*Give P values as exact values whenever suitable.*
☒	☐	For Bayesian analysis, information on the choice of priors and Markov chain Monte Carlo settings
☒	☐	For hierarchical and complex designs, identification of the appropriate level for tests and full reporting of outcomes
☒	☐	Estimates of effect sizes (e.g. Cohen's *d*, Pearson's *r*), indicating how they were calculated

Our web collection on statistics for biologists contains articles on many of the points above.

Software and code

Policy information about availability of computer code

Data collection	Abberior Imspector Acquisition & Analysis Software v16.3, TECAN SparkControl, MEGA-X, RosettaScripts, AlphaFold2-multimer
Data analysis	GraphPad Prism 11.0.1, FIJI, PyMOL Version 3.0.1, All used scripts for structure predication and interface energy calculations are available under https://github.com/agknaus/Jatzlau-Trumpp-et-al.-structural-computational-analysis/, https://doi.org/10.5281/zenodo.19291097.

For manuscripts utilizing custom algorithms or software that are central to the research but not yet described in published literature, software must be made available to editors and reviewers. We strongly encourage code deposition in a community repository (e.g. GitHub). See the Nature Portfolio guidelines for submitting code & software for further information.

Data

Policy information about availability of data

All manuscripts must include a data availability statement. This statement should provide the following information, where applicable:

- Accession codes, unique identifiers, or web links for publicly available datasets
- A description of any restrictions on data availability
- For clinical datasets or third party data, please ensure that the statement adheres to our policy

The data supporting the findings of this study are available from the corresponding authors upon request. RNA raw sequence datasets for Gnathonemus petersii, Pantodon buchholzi, Erpetoichthys calabaricus and Anguilla anguilla have been submitted to NCBI/SRA under the BioProject accession: PRJNA1314022. Source Data are provided with this paper.

Research involving human participants, their data, or biological material

Policy information about studies with human participants or human data. See also policy information about sex, gender (identity/presentation), and sexual orientation and race, ethnicity and racism.

Reporting on sex and gender	na
Reporting on race, ethnicity, or other socially relevant groupings	na
Population characteristics	na
Recruitment	na
Ethics oversight	na

Note that full information on the approval of the study protocol must also be provided in the manuscript.

Field-specific reporting

Please select the one below that is the best fit for your research. If you are not sure, read the appropriate sections before making your selection.

[x] Life sciences [ ] Behavioural & social sciences [ ] Ecological, evolutionary & environmental sciences

For a reference copy of the document with all sections, see nature.com/documents/nr-reporting-summary-flat.pdf

Life sciences study design

All studies must disclose on these points even when the disclosure is negative.

Sample size	All experiments were performed at least three times independently. Sample sizes were not predetermined using statistical methods but were chosen based on common practice in the field and prior experience with similar experimental systems.For cell-based and biochemical assays, n represents independent biological replicates (independent experiments), with technical replicates averaged prior to analysis. In ligand surface binding assays (LSBA), up to 10 cells were analysed per biological replicate, and values were averaged to yield a single data point per replicate (n = 3; total up to 30 cells per condition).For zebrafish embryo experiments, a minimum of 13 embryos per condition were analysed per independent clutch, and experiments were repeated across three independent clutches to ensure biological reproducibility. Fractions were calculated per clutch and averaged across clutches.
Data exclusions	In order to test if the used methods report reproducible on ligand binding and signaling competence, human receptor variants were used as control. If human receptors did not exhibit the expected result, the dataset was excluded and the experiment repeated in whole.
Replication	all findings could be repeated atleast three times, due to the nature of the methods, the effect sizes varies in between these n's, but the overall effect could be repeated for all experiments
Randomization	Randomization was not relevant to our study as data analysis was semi-automated using the described FIJI scripts.
Blinding	Blinding was not possible as the investigators performed the experiment and recorded the data using the microscope. Data anaylsis however was semi-automated using the FIJI script described in the method section.

Reporting for specific materials, systems and methods

We require information from authors about some types of materials, experimental systems and methods used in many studies. Here, indicate whether each material, system or method listed is relevant to your study. If you are not sure if a list item applies to your research, read the appropriate section before selecting a response.

Materials & experimental systems

n/a	Involved in the study	
[ ]	[x]	Antibodies
[ ]	[x]	Eukaryotic cell lines
[x]	[ ]	Palaeontology and archaeology
[ ]	[x]	Animals and other organisms
[x]	[ ]	Clinical data
[x]	[ ]	Dual use research of concern
[x]	[ ]	Plants

Methods

n/a	Involved in the study	
[x]	[ ]	ChIP-seq
[x]	[ ]	Flow cytometry
[x]	[ ]	MRI-based neuroimaging

Antibodies

Antibodies used	Primary antibodies: anti-Halo (ProMega; #G9211; monoclonal mouse antibody), anti-pSMAD2 Ser465/467 (Cell Signaling; #3108; monoclonal rabbit antibody), and anti-GAPDH (Cell Signaling; #2118; monoclonal rabbit antibody) were used at a 1:1000 dilution in 3% w/v BSA/ TBS-T solution. For HRP-based detection, membranes were incubated with secondary goat-α-mouse or goat-α-rabbit IgG HRP conjugates (± 0.8 mg/ml, Dianova; #111-035-144, #115-035-068) at a 1:10000 dilution.
Validation	Halo antibody specificity was confirmed by transient expression of Halo-tagged BMPRs without any detection of crossreactivity. The antibodies have also been validated by the respective companies, https://www.cellsignal.com/products/primary-antibodies/gapdh-14c10-rabbit-monoclonal-antibody/2118 , https://www.cellsignal.com/products/primary-antibodies/phospho-smad2-ser465-467-138d4-rabbit-monoclonal-antibody/3108 , https://www.promega.de/products/protein-detection/primary-and-secondary-antibodies/anti-halotag-monoclonal-antibody/?catNum=G9211

Eukaryotic cell lines

Policy information about cell lines and Sex and Gender in Research

Cell line source(s)	COS-7 cells and HEK293t were obtained from the German Collection of Microorganisms and Cell Cultures (DSMZ). https://www.dsmz.de/collection/catalogue/details/culture/ACC-305<br>https://www.dsmz.de/collection/catalogue/details/culture/ACC-60
Authentication	None of the cell lines used were authenticated.
Mycoplasma contamination	All cell lines tested negative for mycoplasma contamination.
Commonly misidentified lines<br>(See ICLAC register)	No commonly misidentified lines were used.

Animals and other research organisms

Policy information about studies involving animals; ARRIVE guidelines recommended for reporting animal research, and Sex and Gender in Research

Laboratory animals	wild type zebrafish, 22hpf , Strain DBSWT zebrafish, source Department of BiologicalSciences (DBS), National University of Singapore. Wild type zebrafish embryos were obtained by crossing corresponding adult male and female fish.
Wild animals	Our research complies with all relevant ethical regulations; the relevant animal protection committee of the Leibniz-IGB and the city of Berlin (LaGeSo) approved the animal use protocol where relevant. Anguilla anguilla and Cyprinus carpio were kept under animal husbandry permit ZH114 (LaGeSo, Berlin) at IGB; three other fish species (Gnathonemus petersii, Pantodon buchholzi, Erpetoichthys calabaricus, adult) were obtained from commercial dealers and humanely euthanized using an overdose of buffered Tricaine PHARMAQ 1000 MG/G (MS222; concentration: 500 mg/L), for commercial fish at the day of arrival at IGB. This ensured that no pain, suffering, distress or lasting harm was inflicted on the animals.
Reporting on sex	na
Field-collected samples	na
Ethics oversight	Our research complies with all relevant ethical regulations; the relevant animal protection committee of the Leibniz-IGB and the city of Berlin (LaGeSo) approved the animal use protocol where relevant. Anguilla anguilla and Cyprinus carpio were kept under animal husbandry permit ZH114 (LaGeSo, Berlin) at IGB; three other fish species (Gnathonemus petersii, Pantodon buchholzi, Erpetoichthys calabaricus) were obtained from commercial dealers and humanely euthanized using an overdose of buffered Tricaine PHARMAQ 1000 MG/G (MS222; concentration: 500 mg/L), for commercial fish at the day of arrival at IGB. This ensured that no pain, suffering, distress or lasting harm was inflicted on the animals. RNA samples of Xenopus laevis were obtained from control groups of an animal experiment approved by the German State of Health and Social Affairs (LaGeSo, Berlin, Germany; G0359/12).<br>Zebrafish experiments were performed in accordance with protocol BR22-1497 approved by the Institutional Animal Care and Use Committee (IACUC) of the National University of Singapore. Adult zebrafish were housed in recirculating aquaria systems at 28°C under a 14 hour/10 hour light/dark cycle in the fish facility of the Department of Biological Sciences (DBS) at the National University of Singapore.

Note that full information on the approval of the study protocol must also be provided in the manuscript.

Plants

Seed stocks	na
Novel plant genotypes	na
Authentication	na
